# The mental health impact on women of engaging men in health interventions in low- and middle-income countries: A systematic review

**DOI:** 10.1371/journal.pgph.0005168

**Published:** 2025-11-03

**Authors:** Anvita Bhardwaj, Lena Schulhofer, Jenna M. Ledbetter, Joesph J. Gallo, Sarah M. Murray

**Affiliations:** Department of Mental Health, Johns Hopkins Bloomberg School of Public Health, Baltimore, Maryland, United States of America; McGill University, CANADA

## Abstract

The field of women’s health, specifically maternal and child health, have recently pushed for male partners to be included in interventions. Currently, there are gaps in understanding how engaging men in these interventions might impact women’s mental wellbeing. The objective of this systematic review was to examine the evidence of the mental health impact on women of engaging male partners in health interventions in low and -middle-income countries. We conducted a systematic review of existing literature on women’s health interventions that engage male partners and report mental health outcomes at the end of the intervention. The protocol for this systematic review is registered with the PROSPERO database of systematic reviews (CRD42023450412). A tailored search strategy was conducted for both peer-reviewed publications and grey literature. Fourteen peer-reviewed full-text articles fulfilled the inclusion criteria and their quality was appraised. No grey literature fulfilled the inclusion criteria. Studies were compared on key elements of the 1) intervention, 2) men’s engagement methods and measurement, and 3) reported and assessed women’s mental health outcomes. Studies engaged male partners in a variety of ways, including separate concurrent interventions for men and women, joint interventions in which couples went to the intervention sessions together, and a mix of both joint and concurrent intervention components. The majority of studies measured men’s engagement by taking attendance. This systematic review presents critical insights into how men are engaged in women’s health interventions and its impact on women’s mental health. There is a dearth of research on this topic and most interventions only measure men’s engagement programmatically through taking attendance.

## Introduction

Men’s engagement in women’s health interventions has gained increasing recognition by the public health community as a critical implementation strategy for improving health outcomes of both female and male partners [1,2]. The Interagency Gender Working Group defines men’s engagement as “the intentional inclusion of men and boys in family planning problems throughout life stages as supportive partners, contraceptive users, and agents of change [3].” This approach also includes addressing harmful gender norms and power differentials and promoting equitable caregiving, division of labor, and decision-making within families [4]. Though this definition is specific to family planning, men’s engagement is now being applied more broadly across women’s health interventions, including mental health programming [4]. The main goal of this systematic review is to understand the current state of the literature focused on men’s engagement interventions in LMICs and their impact on women’s mental health along with identifying gaps in the research. Currently, there is no review of the literature focused on identifying this relationship. By identifying how different interventions engagement men and potential impact women’s mental wellbeing, future interventions can avoid engaging men in ways that are potentially harmful to women’s mental wellbeing.

Prompted by the 2030 Agenda for Sustainable Development and other global commitments to move beyond simply acknowledging gender disparities in health, the global health sector has shifted from emphasizing gender-neutral to gender-sensitive and gender-*transformative* approaches [5]. Gender-transformative approaches deliberately engage men and women in programming to challenge and change harmful gender norms, relations and structures, thus reshaping inequitable gendered systems that differentially affect women’s health outcomes [5,6] Although measuring the sustainability of social changes and related-health impacts has proved challenging [5]. Evidence suggests that such approaches are promising for advancing gender equity and improving indicators related to sexual and reproductive health, violence, and HIV for women, men, and children [7]. The primary pathways hypothesized to lead to positive health outcomes are: dismantling gender imbalances and inequities; improving men’s understanding and feelings of the importance of women’s health issues; enhancing communication and collaborative and equitable decision-making [6,8–13].

Overall, the empirical evidence of the impact of men’s engagement on women’s health in general is mixed. The culture, context, health related outcome of interest, and framing of why it is important to engage men play a large role on whether the impact of men’s engagement is helpful or harmful [10,14–16]. Aspects such as limited open communication between spouses and late accompaniment to health appointments have been identified as key indicators that men’s engaging in the intervention will adversely impact the women’s health outcome of interest [16]. Most evidence points to engaging men in women’s health interventions as having a positive impact on women’s physical and mental health if done in a gender transformative intervention [10,14,15,17–20]. A prime example of this is the Bandebereho study in Rwanda that implemented a gender transformative couples’ intervention that was shown to have a positive impact on increasing women’s wellbeing [8].

Yet, existing studies have also identified a few pathways of concern. Men involved in maternal and reproductive health interventions have reported increasing their controlling behaviors, such as choosing when to use contraceptives and telling their female partner who she can spend time with and experiencing these kinds controlling behaviors has been linked to increased depression for women [21–24]. If an intervention does not address pervasive gender inequities, studies have found that men are likely to continue to assert control over women during the intervention, impinging upon women’s autonomy to make health decisions and potentially continuing to perpetuate violence [25,26]. Even in interventions that are gender transformative and seek to address gender inequities, research has shown that some men react to these interventions by attempting to assert control in the household [21,27]. Previous studies have shown that inviting men to talk about gender-based violence and toxic masculinity can cause tension in the sessions between men and women [28,29]. In the field of HIV prevention, women have reported being reluctant or scared to involve their HIV-negative partner in interventions due to fear of domestic violence, stigma, and divorce [30–32]. Though many interventions do not delve into what exactly generates that tension or the reasons behind it, one hypothesis is that when couples are forced to confront and acknowledge deeply ingrained gender inequities, the status quo of roles and responsibilities of men and women in the relationship are disrupted [33]. The hesitation and potential negative ramifications of engaging male partners that have been expressed by women could lead to detrimental health outcomes, undermining or complicating the potential benefits of men’s engagement. It is possible to extrapolate potential mechanisms from current literature for how engaging men might generate negative effects on women’s mental health, yet no literature has directly addressed the potential for unintended negative women’s mental health consequences of men’s engagement in women’s health.

The World Health Organization’s (WHO) recommendations on Health Promotion Interventions for Maternal and Newborn Health explicitly advised that men’s engagement strategies need to make sure they are not reducing women’s decision making autonomy and suggested this can be done through avoiding reinforcing traditional gender norms [34]. A recent systematic review found that in practice, only 8% of global interventions that engage men challenge harmful masculinities or unequal power privileges that men have over women [35]. Harmful masculinities and upholding men’s power over women have been shown to increase the likelihood of male-perpetrated violence against women, men’s control over women’s healthcare decision-making making, and men’s lack of involvement in child care, all of which have been linked to increased depression and anxiety in women [35–38]. Thus, most interventions engage men without addressing the underlying, gender norms and power dynamics within relationships, potentially increasing the risk of poor mental well-being for women. Although studies have shown that men’s engagement in women’s health interventions adversely affects women’s empowerment and -decision-making autonomy, the downstream direct impact on women’s mental health has not been studied [39,40].

One of the primary gaps in current evidence is the lack of a standardized way to measure men’s engagement, which contributes to difficulties in understanding the potential mental health impacts of engaging men in women’s health interventions [39,41]. Most studies use programmatic monitoring and evaluation data to assess men’s engagement, relying on tools such as attendance logs, session observation forms, and participant satisfaction surveys [42]. However, none of these methods effectively measure active engagement itself, which has been shown to predict outcomes more robustly than mere attendance [43]. Conventional measures of men’s engagement itself are generally biomedical and do not capture the full range of men’s engagement in women’s health in specific contexts [44,45]. When intervention studies restrict men’s engagement to the biomedical approach, they risk discounting other types of meaningful support [44]. When thinking through measuring men’s engagement, it is important to first assess men’s own understanding and definition of involvement in women’s health and contextualize the measure of engagement to fit social and cultural norms in that specific setting [44,45]. The complexity and cross-cultural differences related to the conceptualization of men’s engagement points is why Galle et al (2021), proposes a comprehensive set of measures instead of a standardized one [46].

The second gap in the literature is the lack of a comprehensive understanding of how men’s engagement actually influences women’s mental health. This, coupled with the absence of standardized measures for men’s engagement across different interventions, complicates the analysis of the relationship between men’s engagement and women’s mental health [10]. To address these gaps, a systematic approach to understanding how men’s engagement is measured and integrated into women’s health interventions that measure a women’s mental health outcome is necessary. Therefore, this systematic review aims to examine the evidence of the mental health impact on women of engaging male partners in health interventions in low- and middle-income countries (LMICs). We chose to focus this review on LMICs and exclude high-income countries (HICs) due to significant culturally and contextually differences such as differences in common gender norms in LMIC versus HIC settings (e.g., women are more commonly part of the workforce in HICs, which may not be the case in many LMICs) [47].

## Methods

The protocol for this systematic review is registered with the PROSPERO database of systematic reviews (https://www.crd.york.ac.uk/prospero/display_record.php?RecordID=450412); registration number CRD42023450412. The PRISMA checklist can be found in the Supplemental Materials (S1 Checklist).

### Search strategy

Searches of keywords, titles, topics, and abstracts were conducted on the following databases for both peer-reviewed publications and grey literature: Web of Science, PubMed, EMBASE, PsychInfo, CINAHL, SCOPUS, WHO Global Index Medicus, IBSS, Cochrane, ProQuest Dissertation and Theses Global, Global Health Observatory (GHO), The Communication Initiative Network, USAID DEC, UK Department for International Development, OAlster, Clinicaltrials.gov, International and Clinical Trial Registry Platform. Search terms were separated into four concepts: (1) Women’s Health, (2) Men’s engagement, (3) LMICs, (4) Gender-Based Violence and Interpersonal Violence. We searched for 1 AND 2 AND 3 NOT 4. Search strategies used for each of the databases can be found in the supplemental materials and were restricted to articles written in English (S1 Text). We also looked through the reference lists of included studies for additional articles. The review covered publications up to August 28, 2024.

### Selection criteria

Duplicates were removed by Covidence, a web-based collaboration software that allows researchers to streamline the production of systematic reviews [48]. Studies were ineligible for inclusion in the systematic review if they: (a) did not take place in a LMIC defined by the World Bank, (b) did not have an intervention, (c) did not focus on a women’s health issue, (d) did not engage male partners, (e) did not report women’s mental health as an outcome and (f) were an intervention that focused solely on IPV or GBV. An intervention was defined as “an organized set of means implemented in a specific context to meet one or several targets with respect to improving health and preventing disease.” [49] We used the women’s health definition from Peters et al., (2000) which redefines women’s health as both health issues that are unique to women such as menstruation and pregnancy, but also health issues that might affect women differently including non-communicable diseases such as cardiovascular disease [50]. Thus, for this review, we included studies for any disease state as long as the sample for the intervention was only women [50]. Mental health was defined as symptoms of emotional distress or an established disorder in the fifth edition of the Diagnostic and Statistical Manual of Mental Disorders (DSM) [51].

Screening took place in two phases: initial title abstract screening and review of full texts of eligibility for inclusion. Three authors were involved in the title and abstract screening process (AB, LS, and JML). All titles/abstracts were double screened such that AB screened all titles and abstracts with LS or JML, blinded to what AB had voted, being the second vote. If there was disagreement between AB and LS or JML, whoever did not originally vote would make the deciding decision and were not blind to how AB and the other screener had voted. If it was unclear if a reference met the inclusion criteria (both voters could not tell), it was included and taken forward into the next stage of the screening process. Full texts of the remaining studies were then examined for eligibility and inclusion by AB and LS. If AB and LS disagreed on any of the full texts SM and JG reviewed and were the deciding vote.

### Quality assessment and data extraction

One reviewer (AB) used the Joanna Briggs Institute (JBI) critical appraisal tools to evaluate the risk of bias for randomized controlled trials and quasi-experimental studies [52]. Data from the included papers were extracted using Covidence by both AB and LS. Extracted data included details on the setting of the intervention, study design, details on the intervention, sample size, primary women’s health outcome, ways male partners were engaged, if and how men’s engagement was measured, and how women’s mental health was measured as an outcome and what the results were for the extracted text (Tables 1–3). Both AB and LS extracted data from each article. If there was disagreement on what to extract AB and LS would meet and discuss.

**Table 1:** Characteristics of the included studies (N = 14).

Citation - 1st author’s last name and year	Country	Setting	Study Design	Sample Size for the Men’s engagement Component of Intervention	Intervention is Delivered By	Type of Intervention	Focus of Intervention/ Intervention’s Content	Duration of Intervention	Location of Intervention	Primary Women’s Health Outcome
**Akbarian 2018**	Iran	Urban	RCT	34 couples	not specified	A mental health training program	The training course content included information such as the definition of mental health and its importance during pregnancy, the impact of mental disorders on pregnancy outcome, the role of husbands in the mental health of pregnant women, marital communication skills, problem-solving skills, breathing techniques, muscle relaxation techniques, stages of vaginal delivery, pain-relief methods, physiological delivery and readiness for accepting the role of parents, and newborn care and breast feeding.	4 training sessions were held during 4 weeks for 2 hours each	Clinical (health center)	Depression, Anxiety and Stress
**Çömez 2020**	Turkey	Urban	Quasi-experimental study	83 couples	Online	Web-based educational training	Quality of life for women with breast cancer and spousal adjustment. The website contained information which was needed by the patients from the diagnosis to the rehabilitation process. The content of the information was about the structure of the breast, breast cancer and treatment methods, the prevention and management of the symptoms that are related to the treatment, sexuality, pregnancy and healthy life recommendations were also provided here	3 months	Clinical and Online (university hospital and online)	Breast Cancer
**Comrie-Thomson 2022**	Zimbabwe	Rural	cRCT	515 couples, and 375 individual women	Trained local female village health workers delivered the women’s intervention. A trained male Organization for Public Health Interventions and Development (OPHID) staff member who was also a nurse and midwife delivered the men’s intervention	Community-based educational trainings and focus group discussions	Coparenting information, equitable maternal and child health decision making and men’s practical support for women and babies.	Monthly 1 hour discussion groups	Community (non-clinical setting)	Postnatal Depression
**Dehshiri 2023**	Iran	Urban	Quasi-experimental study	33 couples	Researcher	Education	Couple’s intimacy and postpartum blues	3 prenatal care sessions and 3 virtual training sessions	Clinical and Online (health centers and online)	Postpartum Blues
**Fourianalistyawati 2023**	Indonesia	National	RCT	8 couples	Online	Mindfulness and educational content	Mindfulness with additional education content aims to reduce stress and fear of childbirth and improve mindfulness in parenting	3 hours a week for 9 weeks, one silent retreat day for 6 hours between class 6 and class 7 and a reunion class between 4–12 weeks after all women have given birth	Online (Zoom)	Depression
**Jones 2018, Jones 2021, Abbamonte 2021, Peltzer 2020 - Peltzer 2020, Sifunda 2019**	South Africa	Rural	cRCT	222 couples, and 122 individual women	Lay healthcare workers	Behavior change intervention and counseling	The PMTCT intervention was based on “Protect Your Family” (PYF), a manualized, behavioral intervention provided by lay healthcare workers. The intervention specifically targets PMTCT, partner HIV testing, ART adherence, HIV stigma, family planning, partner communication, IPV reduction, safe infant feeding, safer conception, HIV serostatus disclosure, and dual method sexual barrier use. The men’s PYF program covered PMTCT for women, men’s issues, and child health issues.	3 prenatal weekly 2 hour group sessions, one prenatal couples counselling session, 2 postpartum couples counselling sessions	Clinical and Community (community health centers)	Prevention-of-mother-to-child-transmission (PMTCT) of HIV/AIDS
**Maitra 2017**	India	Urban	RCT (qualitative results reported)	89 couples attended all the intervention sessions of which 14 couples were interviewed for this manuscript	Facilitators, with master’s degrees in social work and related graduate training, were provided extensive training, regular refresher training, and weekly consultation from project PIs	Narrative intervention method and counseling	The couples’ intervention addressed the psychological and sociocultural factors related to the sexual health concerns of both women and the couple.	6 weeks in total with 2 hour sessions each week	Clinical and Community	Women’s Sexual Health
**Mindry 2018**	Uganda	Rural	Non-experimental one arm study	42 women and 16 male partners	HIV counselors at the clinic	Counseling and education	Safer contraception practices	6 monthly sessions	Clinical (AIDS Support Organization (TASO) clinic)	Contraception Practices
**Mosalanejad 2013**	Iran	Urban	RCT	33 Couples	not specified	Spiritual group psychotherapy counseling	Infertility	12 sessions, 2 hours per week for 3 months	Clinical (hospital)	Infertility
**Rabiepoor 2019**	Iran	Urban	RCT	76 couples	Researcher	Counseling	The agenda of the counseling sessions was based on whatever issues the couple was going through. Some issues discussed in the counseling sessions included physiologic changes during pregnancy and after delivery, health problems during pregnancy, type of delivery, postnatal health issues, breastfeeding, childcare, sex in late pregnancy and after childbirth, nutrition, postnatal care, use of contraceptives, postpartum exercises, and support of husbands	5 counseling sessions in total that lasted between 60 and 80 mins	Clinical (health centers)	Postpartum Depression
**Sayari 2022**	Iran	Urban	Quasi-experimental study	44 couples	Researcher who received training of SSE-CP course and got qualified to counseling	Skill-based sexual enhancement counseling program (SSE-CP)	Sexual difficulties and needs for multiple sclerosis patients	six sessions (twice a week) for three weeks	Community (non-clinical setting)	Multiple sclerosis and General Sexual Health
**Sorkhani 2022**	Iran	Urban	RCT	60 couples	Trained midwife	Counseling	combination of psychological training, supportive counseling and cognitive-behavioral counseling focusing on infertility	6, 45-minute sessions twice a wee	Clinical (hospital infertility center)	Infertility
**Sulaiman 2021**	Pakistan	Urban	RCT	212 couples	Online	Educational	The intervention focuses on developing conflict management, problem solving, communication, and mutual support to foster positive joint parenting of an infant	eight 45-minute online videos, couples were instructed to watch one video a week	Online	Postpartum Depression
**Villar-Loubet 2013**	South Africa	Rural	RCT (qualitative results reported)	119 couples	Trained lay counsellors	Building cognitive behavioral skills	The sessions emphasized cognitive behavioral skills building to improve communication and sexual risk reduction. The intervention focused on couples’ behavioral HIV-risk reduction and medication adherence	four weekly 90–120 min sessions	Clinical (antenatal clinics)	Prevention-of-mother-to-child-transmission (PMTCT) of HIV/AIDS

**Table 2:** Characteristics of how men’s engagement was implemented and assessed in the included studies.

1st author’s last name and year	Strategy for engaging male partners in the intervention	Assessed impact of men’s engagement on the primary women’s health outcome through having an arm with or without men’s engagement	Findings on the impact of men’s engagement on the women’s health outcome	Assessed degree of men’s engagement
**Akbarian 2018**	Participants were randomly divided into three groups one of which was couples (with the presence of the partner). Husbands attended the training sessions	Yes	Analyses showed that the couples’ group had the lowest anxiety and stress scores of all three, and a tied for lower depression score with the women’s only intervention group.	No
**Çömez 2020**	Participants were couple dyads, spouses had access to the website and were allowed to write questions, receive answers and view content	No	N/A	No
**Comrie-Thomson 2022**	Men (coparents) participated in the second intervention, + Men, which involved one monthly one-hour group discussions, facilitated by the male project staff member in men’s workplaces or a central community location.	No	N/A	No
**Dehshiri 2023**	Men accompanied their female partner to 3 of 6 prenatal sessions in the intervention arm and then participated in the virtual trainings	No	Husband involvement may be the reason for significant increase in the mean changes in postpartum blues in the intervention group compared to the control group. The findings of the present study showed that the incidence of postpartum blues was significantly lower in the intervention group than in the control group.	Yes, using men’s attendance
**Fourianalistyawati 2023**	Participants were couple dyads and concurrently went through intervention, they were instructed to be in the same room and join the online sessions together	No	N/A	Yes, using men’s attendance
**Jones 2018, Jones 2021, Abbamonte 2021, Peltzer 2020, Sifunda 2019**	Male partners participated in the intervention. The intervention sessions for men addressed PMTCT for women, men’s issues, and child health issues, and were designed to be appropriate to partners of both HIV+ and HIV− women.	Yes	Male involvement was a protective factor against depressive symptoms, though women experiencing psychological intimate partner violence (IPV), HIV stigma, or having less education, had greater depressive symptomatology”	Yes, using the Adapted Male Involvement Index
**Maitra 2017**	Couples engaged in group counseling sessions. The first four sessions were single-gender groups with wives and husbands meeting separately and then sessions 6 and 6 were mixed gender sessions.	Yes	Some husbands were reluctant to attend, despite repeated follow-up visits. While most women were positive about participation, when the men did not attend the sessions, neither could their wives. Thus, if men did not attend women were not able to receive the intervention that potentially could help the main outcome of interest in this study which was women’s sexual wellbeing.	Yes, using men’s attendance
**Mindry 2018**	Couples attended the safer-conception counseling sessions together	No	N/A	No
**Mosalanejad 2013**	Attended the group logotherapy sessions as a couple together	No	N/A	No
**Rabiepoor 2019**	Couples attended the intervention sessions together except for session #3 which featured only fathers	No	N/A	No
**Sayari 2022**	Couples attended the sessions together	No	The impact of men’s engagement was discussed in the article more so as a mechanism that positively impacted the general sexual health wellbeing for women living with multiple scleoris. For instance, after explaining multiple sclerosis patients shared sexual problems, the spouses showed higher compassionate sensitivity to their spouse. They hypothesize that intervention is effective because it creates constructive intimacy and a healthy atmosphere for couples to emotionally express themselves and cooperate to solve their sexual and psychological issues.	Yes, using men’s attendance
**Sorkhani 2022s**	Couple-dyads in both intervention and control groups received counseling together	No	The authors hypothesized that anxiety scores may not have improved after counseling as consistent with previous studies because participants were couples, not individual women. They also did not specify whether the women filled out the questionnaires in the presence of their husbands or on their own.	Yes, using men’s attendance
**Sulaiman 2021**	Participants were couple dyads and concurrently went through intervention by watching the videos together and going through the interactive activities and activity-based coparenting workbook together	No	Of the 60 couples who watched some or all of a full video in the intervention, 43 couples watched the videos together, 9 watched at times that worked for each individual, and 8 couples did not specify whether they watched the video together or alone. Follow-up participants who did not complete the questionnaire were more likely to have a partner of Muhajir ethnicity and/or working in the private sector, suggesting partners were a variable impacting adherence. Couples filled out forms together and impact of partner involvement not discussed.	Yes, attendance/ participation was measured through tracking when a couple watched the online videos
**Villar-Loubet 2013**	In parallel gender-concordant group sessions	No	During role-plays regarding partner failure to attend appointments, women responded with a range of emotions, such as ‘angry’, ‘hurt’ and ‘disappointed.’ Women often perceived men as apprehensive and uninterested in attending clinic appointments. Although some men might accompany their partner to an appointment, they usually wait in the clinic area instead of entering the consulting room with their partner. Alternately, women indicated the group intervention had a positive impact on partner involvement, including increasing the number of times they accompanied them to the clinic and on the quality of their communication (e.g.,., increasing effective communication).	Yes, using men’s attendance

**Table 3:** Characteristics pertaining to women’s mental health as an outcome of interest for the intervention.

Citation - 1st author’s last name and year	Women’s Mental Health Outcome of Interest (all are symptoms of disease)	Tool Used to Measure Women’s Mental Health/ Substance Use Outcome	Mental Health Results for Women at the End of the Intervention
**Akbarian 2018**	Depression; Anxiety; Stress	Depression, Anxiety, and Stress Scales (DASS-42)	The decrease in means scores pre-post intervention for anxiety and stress in the couples arm was significantly lower than the only women intervention arm and control arm, but change in depression was not significantly different between the two intervention arms.
**Çömez 2020**	Emotional well-being	Emotional well-being 6-item sub scale of functional assessment of cancer therapy-breast cancer (FACT-BC) scale	Women’s emotional well-being significantly improved from baseline to the end of the intervention and was significantly higher than the emotional well-being scores of the control group at the 3 month mark.
**Comrie-Thomson 2022**	Depression	Edinburgh Postnatal Depression Scale (EPDS)	Women’s EPDS scores declined significantly more in the couples based intervention arm than the non-couple’s based intervention arm. The decline was 34% greater in the intervention arm (adjusted risk ratio = 0.66; 95% confidence interval = 0.48, 0.90, P = 0.008). A decline in EPDS scores means that women displayed less postnatal depression symptoms which is a positive change.
**Dehshiri 2023**	Postpartum blues	Stein’s postpartum blues questionnaire	The results showed that 5 women (15.20%) in the intervention group and 26 (72.20%) in the control group suffered from postpartum blues after intervention (p < 0.001).
**Fourianalistyawati 2023**	Depression	Edinburgh Postnatal Depression Scale (EPDS)	Women in the intervention group experienced a greater reduction in depressive symptoms compared to the treatment as usual group.
**Jones 2018, Jones 2021, Abbamonte 2021, Peltzer 2020 - Peltzer 2020, Sifunda 2019**	Depression	The Edinburgh Postnatal Depression Scale 10 (EPDS-10)	Women in phase 2 where they had their partners present were less depressed as seen by the decline in EPDS-10 scores.
**Maitra 2017**	General emotional distress; Other: Tension	Qualitative Methods (In-depth interviews)	Both male and female participants reported reduction in their “tension” levels.
**Mindry 2018**	Anxiety; General emotional distress	Qualitative Methods (In-depth interviews)	Attending counseling sessions together was described as an important aspect of maintaining good health, including mental health. One woman specifically mentioned that attending counseling sessions together minimized her anxiety.
**Mosalanejad 2013**	Other: stress and worry	Penn State Worry Questionnaire and Perceived Stress Scale PSS 14	Both the PSWQ and PSS significantly decreased more in the treatment group compared to control
**Rabiepoor 2019**	Depression	The Edinburgh Postnatal Depression Scale	The mean EPDS scores 2 months and 6 months after childbirth were significantly lower in the intervention groups compared to the control pointing to a reduction in postnatal depression symptoms.
**Sayari 2022**	Other: Quality of life	54-item MS Quality of Life Questionnaire (MSQOL-54)	The intervention group had significantly higher scores for the psychological quality of life scale at the post-test and follow up periods.
**Sorkhani 2022**	Depression; Anxiety; General emotional distress	Screening on Distress in Fertility Treatment (SCREENIVF); Spielberg Questionnaire (1–10);	There was a significant decrease in depression in the intervention group compared to the control group, but there were no significant differences in change in anxiety
**Sulaiman 2021**	Depression; Anxiety; Other: Postpartum depression	Edinburg Postnatal Depression Scale (EPDS); State-Trait Anxiety Inventory (STAI)	No statistically significant findings but the intervention did increase the absolute risk of anxiety symptoms and relative risk of anxiety for women.
**Villar-Loubet 2013**	General emotional distress, depression, stress	Qualitative Methods	Participants found that the intervention helped them with dealing with anger and relieve stress.

## Results

**Fig 1:**
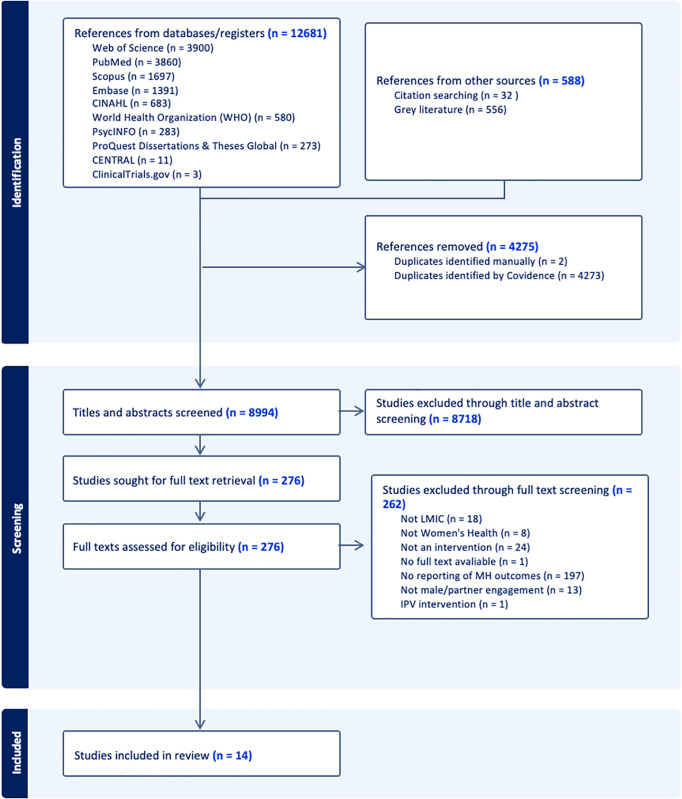
PRISMA diagram of Screened and Included References.

The PRISMA diagram below (Fig 1.) details the selection process. The literature yielded 8,994 (S2 Table) unique references of which 8,718 (S3 Table) were excluded in the title and abstract screening step due to the following exclusion reasons; (1) not being set in a LMIC, (2) not having a women’s health outcome, (3) not an intervention, (4) did not report mental health outcomes at the end of the intervention, (5) did not have a men’s engagement component to the intervention, (6) was an intervention focused in violence. 276 full texts (S4 Table) were assessed for eligibility and 262 were excluded leaving 14 studies to be included in this review. All of the exclusion criteria were assessed at each step to arrive at the 14 included studies. The 14 studies were discussed between two authors (AB and LS) to confirm they were eligible to be included in the study. Five articles, [53–57], reported on the same intervention called ‘Your Family’ and were thus grouped and counted as one study, leaving 14 unique studies. There was no missing data from the included studies.

### Study characteristics

Key details of each study are presented in Table 1. Twelve studies were published, peer-reviewed journal articles and two were dissertations. Six studies were conducted in Iran [58–63], two in South Africa [53,64], and one each in Turkey [65], Zimbabwe [66], Indonesia [67], India [68], Pakistan [69] and Uganda [70]; the majority of studies (n = 9) were conducted in an urban region. Most studies used a randomized controlled design (n = 10), three a quasi-experimental design, and one a non-experimental one-arm study to evaluate the impact of an intervention with pre- and post-measures and no control group. Two studies that were RCTs reported qualitative results, with one reporting results for the intervention and control arm separately, [68] and one only reported the qualitative results from the intervention arm [64]. More detail about the types of interventions can be found in the section below, but the interventions ranged from online educational trainings focused on the women’s health outcome that were then also offered to men to interventions specifically adapted to be offered to the couple such as a mindfulness intervention to reduce stress for new parents. The sample size in these studies varied from 515 [66] to 8 couples in Fourianalistyawati (2023) [67]. Studies were published between 2013 and 2023. Fourianalistyawati (2023) and Sulaiman (2022) were both dissertations for a Doctor of Philosophy degree [67,69].

All studies were assessed as being high quality based on the JBI Critical Appraisal Checklists. The results of the quality appraisal can be found in the Supplementary Materials (S1 Table). For the ten RCTs, the main source of risk of bias arose from the inability to blind the participant and the outcome assessor on the treatment assignment, as studies often compared a treatment-as-usual control group to an intervention arm that purposefully included male partners.

### Interventions

#### Methods and content of intervention.

Most of the interventions included in the review had an educational component or a counseling component. The primary outcome evaluated following the interventions for women in six studies were symptoms of a mental health disorder. Comrie-Thomson (2022), Dehshiri (2023), Rabiepoor (2019) and Sulaiman (2022) all described interventions that were focused on postpartum depression or postpartum blues [59,62,66,69]. The other two studies [58,67] focused on common mental disorders (depression and anxiety) that were not tied to the postpartum period. Five studies focused on women’s reproductive health issues including infertility [61,63], prevention-of-mother-to-child transmission of HIV/AIDS (PMTCT) [53–57,64], and contraception practices [70]. Maitra (2017) focused on women’s sexual health through an intervention that addresses the interactional dynamics within a married couple and specifically focused on HIV and STI risk and prevention [68]. The remaining two studies focused on physical health issues: Çömez (2020) [65] focused on breast cancer and Sayari (2022) [60] focused on Multiple Sclerosis (MS) along with general sexual health for women.

#### Setting.

Most studies (n = 10) took place in a single setting, with six studies occurring in clinical settings (i.e., hospitals and clinics) [58,61–64,71], two in community settings that were non clinical (i.e., a non-profit organization’s office and a central community location that was not specified), and two online [67,69]. Four studies took place in two locations: two delivering the intervention in a clinical and digital space [59,65] and the other two in a clinical and community setting [53,68]. Most commonly, the tested intervention was delivered by trained lay health professionals or a research team staff member. Two studies did not specify who was delivering the intervention [58,61].

#### Outcomes of the intervention.

Almost all interventions included in the systematic review had a health education component of the intervention; in six studies this education was primarily focused on the targeted women’s health outcome. For example, in Fourianalistyawati (2023) [67], a mindfulness intervention was supplemented with education on childbirth with the aim that the educational component would help reduce the stress the couple was feeling. Education often focused on something the couples were doing collaboratively such as parenting in Sulaiman (2021) [69], or deciding on contraceptive practices in Mindry (2018) [70]. In Cömez (2020)[65], a web-based educational intervention, the educational components primarily focused on quality of life for women with breast cancer, covering symptom management, treatment timelines, and spousal adjustment. Counseling was used in half of the studies as an intervention strategy (n = 7) [53,60,61,63,68,70,72]. The counseling interventions varied between studies and included individual, couples-based, and group sessions. Jones (2018) and the “Protect Your Family” intervention is an example of an intervention where a mix of counseling types was used (single-gender group sessions and couples counseling sessions) [53]. In contrast, Rabiepoor (2019) [62], allowed couples to build the agenda of the counseling sessions based on current relational issues. Some issues that were discussed in the counseling sessions were physiological changes during pregnancy, postnatal health issues, breastfeeding, and contraceptive use.

### Contrast of men’s engagement methods

The men’s engagement component of most interventions involved both partners being present for and participating in all sessions together. Mosalenejad (2013) [61], for example, conducted 12 group therapy sessions including spiritual and psychotherapy counseling where both partners were present and attended the sessions together. The intervention group met for 2 hours each week for a total of 12 weeks [61]. Rabiepoor (2019) was the only couples-based counseling intervention with a session specifically for men, which occurred 3–5 days after delivery of the baby and was the third session of the intervention [62]. For the online interventions, couples were encouraged to watch the videos or engage in the activities together, but the research team could not identify if men were actively engaged in the intended manner [67,69]. Sulaiman (2022) engaged male partners in an online postpartum depression intervention where they encouraged couples to watch educational videos together and work through interactive activities in a workbook that focused on co-parenting. In Dehshiri (2023) and Jones (2018), male partners accompanied women to some sessions but also had separate intervention components designed specifically and solely for them, e.g., men’s only WhatsApp groups in Dehshiri (2023) and separate men’s only sessions in Jones (2018) [53,59].

### Measurement of men’s engagement

Although all studies had a men’s engagement component in the intervention, six studies did not measure men’s engagement in any manner (see Table 2) [58,61,62,65,66,70]. Seven studies measured men’s engagement via program monitoring [59,60,63,64,67–69], i.e., they captured how many sessions men attended or the frequency of their participation in the intervention, but these studies did not assess the quality of participation, i.e., how actively they were engaging or if they were gaining and/or employing new skills or knowledge. These studies also did not report the rate of missingness of this programmatic data. The five studies on the “Protect Your Family” intervention were the only studies that measured men’s engagement using a scale to assess the quality of men’s engagement [53–58]. Specifically, they adapted the Male Involvement Index and used it as a comprehensive measure of male partner involvement and participation in the intervention [73]. The revised index separated men’s engagement into two types: communication (e.g., discussing antenatal care with your partner) and action-based engagement (e.g., did you attend antenatal care visits with your partner) [73]. Importantly, the Male Involvement Index was administered to both individuals in the couple [73].

### Women’s mental health outcomes

Most studies (n = 8) measured depression as the women’s mental health outcome of interest [53,58,62–67,69]. Other studies looked at anxiety and general emotional wellbeing. Quantitative scales were most often used to measure the mental health outcome of interest. Some of the scales use were the Edinburgh Postnatal Depression Scale (EPDS), Penn State Worry Questionnaire, and the Depression, Anxiety and stress Scales (DASS-42). The other three studies qualitatively measured the women’s mental health outcomes often through in-depth interviews [64,68,70]. In terms of overall effectiveness, the majority of studies (n = 12) reported an improvement in the women’s mental health outcome of interest in the intervention group by the end of the intervention (see Table 3) [53,59–61,63–68,70,72]. Only two studies had null findings or identified an increase in risk or severity of symptoms related to the mental health outcome of interest [58,69]. Though results were not statistically significant, Sulaiman (2021) was the only study that showed an increase in the relative risk of anxiety in the intervention group where men were engaged as compared to the control condition which was treatment as usual [69]. Sulaiman (2022) did not compare the intervention with and without engaging men [69]. Akbarian (2018) included a three-arm study focused on depression, anxiety and stress during pregnancy with a treatment-as-usual control arm, a women-only intervention arm, and second intervention arm with women and men [58]. The men’s engagement intervention arm engaged male partners in a training course that included information on the definition of mental health, importance of mental health during pregnancy, marital communication skills, problem-solving behavior, and other techniques to reduce stress and anxiety during pregnancy. The study saw an overall decrease in depression between both intervention groups and the control group but no significant difference in the decrease of depression between the men’s engagement intervention arm and the women-only intervention arm [58].

### Comparing the intervention with men’s engagement vs only women

Only three interventions [53,58,68], assessed the impact of men’s engagement by comparing the same intervention with and without a men’s engagement component. Maitra (2018) [68], for example, ran a study with four arms: (1) the control arm, (2) women’s individual counseling, (3) group couples counseling, and (4) women’s individual counseling plus group couples counseling [68]. This allowed for the researchers to compare men’s engagement in an intervention to the intervention alone without men’s engagement. They could not compare the magnitude of change between treatment arms through their qualitative analysis. In all three of these studies, women reported decreases in depression and anxiety symptoms and improvement in mental well-being at the end of the intervention compared to the control group of no intervention. In Akbarian (2018), there was no significant difference in reduction in depression, anxiety, or stress observed between the men’s engagement intervention arm and the women’s only intervention arm [58]. For Jones (2018) the men’s engagement intervention was conducted at a later date than the women’s only intervention, and due to unmeasured confounders associated with this time difference, the research team that implemented the intervention and us as the systematic review team cannot compare the women’s only intervention to the intervention arm that engaged men [53].

## Discussion

We performed robust searches of peer-reviewed and grey literature databases that allowed us to identify 14 peer-reviewed articles and manuscripts on women’s health interventions that assessed changes in women’s mental health as an outcome and included a strategy for engaging male partners in the intervention. Studies were compared on key elements of the intervention, men’s engagement methods and measurement, and reported women’s mental health outcomes. Studies engaged male partners in a variety of ways, including separate concurrent interventions for men and women, joint interventions in which couples attended the intervention sessions together, and a mix of both joint and concurrent intervention components. Most studies engaged men in women’s health interventions focused on pregnancy, contraception, infertility, or depression in the postpartum period. Based on the JBI Quality Assessment, the research team or intervention facilitator was never blinded to treatment or control allocation, which increased bias within the studies.

Our findings suggest that there is a dearth of research specifically focused on engaging male partners in women’s health interventions in ways that positively impacts women’s mental wellbeing, effective methods for measuring men’s engagement beyond attendance tracking, and the potential impact of such engagement on women’s mental health, including conditions under which it may be harmful. While this review evaluated interventions that engaged male partners in women’s health programming, the strategies they incorporated did not include gender-transformative programming. The majority of interventions reviewed primarily involved male partners in supportive roles rather than as active participants in programming that seeks to address gender norms and power dynamics as key determinants of women’s health. This gap in the reviewed studies highlights the need to explore the operationalization and specific impact of men’s engagement as a key component of gender-transformative programming on women’s mental health, as well as how women evaluate the impact of different men’s engagement strategies on their mental health. Future studies should not only provide richer descriptions of the nature of male engagement but evaluate how program components directly targeting structural and gender norms (as with gender transformative programming) may differentially effect men’s and women’s relational and health outcomes.

For the studies that attempted to measure men’s engagement in the intervention, only one [53], used a validated scale for men’s engagement as opposed to different sources of measurement such as attendance, or failed to measure men’s engagement at all. Though tracking attendance (usually with a yes/no question) is often used in public health as a proxy for intervention engagement, it does not accurately reflect an individual’s level of attention during sessions, their genuine comprehension the of the content, or the extent to which they can apply learnings to their daily life [74,75]. A recent systematic review looking at web-based interventions similarly highlighted that measuring engagement during online interventions should include metrics beyond just attendance [76,77]. Another review focused on men’s engagement in reproductive, maternal and child health and well-being in East Africa also highlighted the heterogeneity of measurement methods for men’s engagement and the need for more rigorous measurement [41]. Involving male partners in antenatal care visits had a positive impact on the uptake of maternal health services based on a systematic review focused on LMICS [15]. A global framework using five categories (involvement in communication, involvement in decision-making, practical involvement, physical involvement and emotional involvement) for assessing men’s engagement has been developed and is a positive step to standardizing the field, but is only for maternal health and thus will need further refinement to be applicable to all women’s health areas and different cultural contexts [46].

Yet, there are opportunities to characterize engagement more robustly. For example, two of the interventions in our review used online interventions and included attempts to stimulate active men’s engagement. One of the two online interventions mentioned providing worksheets for couples to complete together, but it did not specify if the research team reviewed these worksheets to assess engagement [69]. To monitor men’s engagement more robustly, the research team could have reviewed the worksheets or administered a knowledge-based quiz at the end of them to measure comprehension of the content. The measurement of men’s engagement should go beyond instrumental actions such as attending a counseling session and also include other aspects of involvement such as communication, support and shared decision making [78].

Given our focus on women’s mental health outcomes all interventions had a behavioral health component to them. Cognitive and affective measurement tools to measure engagement and adherence in behavioral health interventions would be important indicators for men’s engagement for the interventions in our study [77]. Other studies recommend that for in-person mental health interventions engagement can be measured by observations, such as noting how often individuals discusses their feelings during a session, the effort they put in, or whether they appear distracted [79]. Some challenges with this approach is that it requires significant human resources and can be time consuming for the person delivering the intervention [77]. The issue of using attendance as a measure of engagement is happening across the field of public health [76,78]. The education sector has looked at the issue of measuring student engagement and recommends a combination of student self-report surveys, teachers’ ratings, observations, attendance and real-time measures such as posting on a class’s discussion forum or using eye-tracking to see how long a student takes to read something online [80]. Overall, engagement measurement can be improved through the use and triangulation of multiple methods.

We limited our review to studies that measured a women’s mental health outcome and thus looked at only a subset of interventions that employ men’s engagement methods to better women’s health. All but one study saw a reduction in women experiencing mental health symptoms by the end of the intervention. This is supported by previous reviews where the authors observe husband’s support and involvement as a protective factor of maternal depression in LMICs and believe this occurs because gender inequality is common in these contexts and thus increased support can boost women’s mental wellbeing [81]. Our systematic review extends these findings in two ways. First as we looked beyond maternal depression and looked at other mental health outcomes including anxiety and depression outside of the peripartum period. Second, we also looked at women’s mental health across the life course including during chronic illnesses such as cancer and multiple sclerosis, infertility and HIV.

Although the studies identified in this systematic review did not look at the long term impacts on women’s mental health, the 6 year follow up from the Bandebereho program showed sustained impact on women’s mental well-being with women reporting fewer depressive symptoms, and improved communication as a couple and can be used by future researchers as a strong example of a gender transformative program that engages men in a way that has a positive impact on women physical health [11]. Improved communication as a couple along with greater trust between the couple are both important potential mechanisms of change strategies that positively impact women’s mental well-being [82,83]. Two studies included in our review had a specific intervention component to improve communication between the couple [58,64]. Shared appraisal of disease and planning of the treatment steps together has also been shown to improve the mental wellbeing of the patient as it increases both instrumental and emotional support from the healthy partner [84,85]. Future studies should look at if there needs to be a specific intervention component addressing a couple’s communication, or if just having men involved in an intervention itself helps improve communication and thus leads to better mental health.

There is also a need for future research to further understand the pathways through which men’s engagement programs may improve women’s mental health. A recent formative research study amongst newlywed women in Nepal showed that better relationship quality with her spouse significantly reduced depression symptoms as did a better relationship with her mother in law [86]. Qualitative research might help understand what women who are the potential participants in the interventions where researchers implement an intervention deem as the most important to improve their mental wellbeing. There is also the importance of understanding variations in culture and context when building men’s engagement interventions. The mental health space can learn from ongoing violence prevention programs that have tackled challenges in implementation due to cultural and contextual differences in sites by working with smaller groups of men to get buy in, addressing the core issues of gender norms in some spaces, and focusing on women’s empowerment as a part of the intervention [87].

Only three studies had intervention arms with and without the men’s engagement component and allowed them to look at the impact of men’s engagement on women’s mental health by comparing the intervention arms, each of these studies showed different results. These findings are supported by broader reviews looking at the global conceptualization of men’s engagement in maternal health and gender transformative interventions where engaging men in maternal mental health interventions in LMICs has been associated with better women’s mental health outcomes [46,88–90]. Given both our findings and ones from previous reviews, we suggest more robust comparison methods to further understand the pathways by which male engagement interventions have better outcomes. This can be done by comparing a women’s health intervention implemented with and without a men’s engagement component, researchers can discern whether differences in the outcome may be attributable to the intervention itself or to men’s engagement. By comparing the intervention being delivered to only women to the intervention having a men’s engagement component the research team could potentially see which of the intervention designs helped the women’s health outcome of interest more.

Thus, one major implication of this review’s findings is that, women’s mental health generally improves in interventions where male partners are engaged. However, in studies comparing men’s engagement interventions to women-only interventions, there is no consensus on which approach is more beneficial for women’s mental health. Along with this lack of consensus in the field, there were a range of scales used to measure mental health, only a few had been validated to use in the setting and population they were used in. Thus, the scales used might not be the most accurate measure of mental wellbeing for that study. In addition to highlighting the need for further research, this finding suggests that the intervention setting, the specific women’s health focus, or the manner of engaging men might have had a positive or negative impact on women’s mental well-being.

Another important shortcoming is that no study reported men’s mental health outcomes. For instance, Suaiman (2022) [69], only assessed women’s mental well-being after an online intervention for postpartum depression, even though studies have highlighted that men can also experience postpartum depression [91]. Rather than a joint intervention for couples, a concurrent single-sex intervention delivered to women and their partners separately might improve postpartum depression by addressing that both partners may face mental health challenges at the same time and being able to focus on the individuals particular feelings at that time [91]. Other studies point to the importance of the quality of male support during the postpartum period, underscoring the need for interventions to educate men on how to best provide support to their female partners [32].

We acknowledge that there are a few limitations to our systematic review. We broadly defined the outcome of women’s mental health to identify as many relevant studies as possible but, along with our inclusion of two qualitative research studies, prevented us from conducting a meta-analysis. We were limited to publications and grey literature written in English, which means we potentially missed relevant literature from many non-English-speaking LMICs. We also did not distinguish between interventions that aim to increase male engagement as an intended outcome and interventions that engage men as an approach (i.e., gender transformative sessions with men) as many of the studies included had both of these components in their intervention. However, since this is the first review looking specifically at the impact of men’s engagement on women’s metal health, we believe that our narrative summary provides an accurate and insightful synthesis of the information across the included studies that can drive future research.

## Conclusion

Male partners play a critical role in women’s health interventions and the field of public health is moving towards increasing men’s engagement in women’s health. This systematic review reveals that engaging men in women’s health interventions in LMICs does not seem to negatively impact women’s mental well-being but that there is a need to conduct robust studies to assess this. We also identified that men’s engagement is most often measured through tracking men’s attendance (yes/no) in sessions and activities). We suggest that future interventions that have a men’s engagement component look at measuring quality of engagement through multiple methods that go beyond attendance, including observation and self-report of level or degree of engagement. Our research emphasizes the importance of understanding how to best engage men in women’s health interventions globally.

## Supporting information

S1 Text Search strategy. (DOCX)

S1 Checklist PRISMA checklist. (DOCX)

S1 Table Quality assessment using the JBI critical appraisal. (DOCX)

S2 Table Title abstract exclusion reasons. (XLSX)

S3 Table Excluded at full text level. (XLSX)

S4 Table Data extraction dates. (XLSX)
