# Supplementary material for: The mental health impact on women of engaging men in health interventions in low- and middle-income countries: A systematic review
Source: PLOS Glob Public Health. 2025 Nov 3;5(11):e0005168. doi: 10.1371/journal.pgph.0005168 (PMC12582469; doi:10.1371/journal.pgph.0005168)
Supplement: S1 Text — (DOCX) [file pgph.0005168.s001.docx]

**Web of Science**

**Concept 1: Women’s Health**

TS=(“women health” OR “reproductive health” OR “maternal health” OR "women's" OR "breast cancer" OR "cervical cancer" OR "obstetrics" OR "gynecology" OR "endometriosis" OR "menopause" OR "osteoporosis" OR "anemia" OR "thyroid" OR "menstrual health" OR "women's health" OR "female health" OR "sexual health" OR "prenatal care" OR “antenatal care” OR "women's screening" OR "women's preventive care" OR “perinatal depression” OR “postpartum depression” OR “postpartum” OR “maternal mental health” )

**Concept 2: Male/Partner engagement**

TS=(“male engagement” OR “husband engagement” OR “partner engagement” OR “male involvement” OR “husband involvement” OR “partner involvement” OR “male participation” OR “husband participation” OR “partner participation” OR “couple based” OR “couple involvement” OR “couple intervention” OR “spouse involvement” OR “spouse participation” OR “couples”)

**Concept 3: LMICS**

**TS=(Deprived Countries OR Deprived Population OR Deprived Populations OR Developing Countries OR Developing Country OR Developing Economies OR Developing Economy OR Developing Nation OR Developing Nations OR Developing Population OR Developing Populations OR Developing World OR LAMI Countries OR LAMI Country OR Less Developed Countries OR Less Developed Country OR Less Developed Economies OR Less Developed Nation OR Less Developed Nations OR Less Developed World OR Lesser Developed Countries OR Lesser Developed Nations OR LMIC OR LMICS OR Low GDP OR Low GNP OR Low Gross Domestic OR Low Gross National OR Low Income OR Lower GDP OR lower gross domestic OR Lower Income OR Middle Income OR Poor Countries OR Poor Country OR Poor Economies OR Poor Economy OR Poor Nation OR Poor Nations OR Poor Population OR Poor Populations OR poor world OR Poorer Countries OR Poorer Economies OR Poorer Economy OR Poorer Nations OR Poorer Population OR Poorer Populations OR Third World OR Transitional Countries OR Transitional Country OR Transitional Economies OR Transitional Economy OR Under Developed Countries OR Under Developed Country OR under developed nations OR Under Developed World OR Under Served Population OR Under Served Populations OR Underdeveloped Countries OR Underdeveloped Country OR underdeveloped economies OR underdeveloped nations OR underdeveloped population OR Underdeveloped World OR Underserved Countries OR Underserved Nations OR Underserved Population OR Underserved Populations Afghanistan OR Albania OR Algeria OR American Samoa OR Angola OR Armenia OR Azerbaijan OR Bangladesh OR Belarus OR Byelarus OR Belorussia OR Belize OR Benin OR Bhutan OR Bolivia OR Bosnia OR Botswana OR Brazil OR Bulgaria OR Burma OR Burkina Faso OR Burundi OR Cabo Verde OR Cape Verde OR Cambodia OR Cameroon OR Central African Republic OR Chad OR China OR Colombia OR Comoros OR Comores OR Comoro OR Congo OR Costa Rica OR Côte d'Ivoire OR Cuba OR Democratic People’s Republic of Korea OR Djibouti OR Dominica OR Dominican Republic OR Ecuador OR Egypt OR El Salvador OR Equatorial Guinea OR Eritrea OR Ethiopia OR Fiji OR Gabon OR Gambia OR Gaza OR Georgia OR Georgia Republic OR Ghana OR Grenada OR Grenadines OR Guatemala OR Guinea OR Guinea Bissau OR Guyana OR Haiti OR Herzegovina OR Hercegovina OR Honduras OR India OR Indonesia OR Iran OR Iraq OR Ivory Coast OR Jamaica OR Jordan OR Kazakhstan OR Kenya OR Kiribati OR Korea OR Kosovo OR Kyrgyz OR Kirghizia OR Kirghiz OR Kyrgyzstan OR Lao PDR OR Laos OR Lebanon OR Lesotho OR Liberia OR Libya OR Macedonia OR Madagascar OR Malawi OR Malay OR Malaya OR Malaysia OR Maldives OR Mali OR Marshall Islands OR Mauritania OR Mauritius OR Mexico OR Micronesia OR Moldova OR Mongolia OR Montenegro OR Morocco OR Mozambique OR Myanmar OR Namibia OR Nepal OR Nicaragua OR Niger OR Nigeria OR Pakistan OR Palau OR Papua New Guinea OR Paraguay OR Peru OR Philippines OR Principe OR Romania OR Rwanda OR Ruanda OR Samoa OR Sao Tome OR Senegal OR Serbia OR Sierra Leone OR Solomon Islands OR Somalia OR South Africa OR South Sudan OR Sri Lanka OR St Lucia OR St Vincent OR Sudan OR Surinam OR Suriname OR Swaziland OR Syria OR Syrian Arab Republic OR Tajikistan OR Tadzhikistan OR Tajikistan OR Tadzhik OR Tanzania OR Thailand OR Timor OR Togo OR Tonga OR Tunisia OR Turkey OR Turkmen OR Turkmenistan OR Tuvalu OR Uganda OR Ukraine OR Uzbek OR Uzbekistan OR Vanuatu OR Venezuela OR Vietnam OR West Bank OR Yemen OR Zambia OR Zimbabwe)**

**Concept 4: Not GBV/IPV**

TS=(“interpersonal violence” OR “gender based violence” OR “violence against women” OR “domestic violence” OR “violence” OR “marital conflict” OR “conflict resolution”)

**#1 AND #2 AND #3 NOT #4**

**PubMed**

**Concept 1: Women’s Health**

"Women's Health"[Mesh] OR "Women's Health Services"[Mesh] OR "Maternal Health Services"[Mesh] OR "Reproductive Health"[Mesh] OR "Reproductive Health Services"[Mesh] OR “women’s” [tw] OR "breast cancer" [tw] OR "cervical cancer" [tw] OR "obstetrics" [tw] OR "gynecology" [tw] OR "endometriosis" [tw] OR "menopause" [tw] OR "osteoporosis" [tw] OR "anemia" [tw] OR "thyroid" [tw] OR "menstrual health" [tw] OR "women's health" [tw] OR "female health" [tw] or "sexual health" [tw] OR "prenatal care" [tw] OR “antenatal care” [tw] OR "women's screening" [tw] OR "women's preventive care" [tw] OR “perinatal depression” [tw] OR “postpartum depression” [tw] OR “postpartum” [tw] OR “maternal mental health” [tw]

**Concept 2: Male engagement**

“male engagement” [tw] OR “husband engagement” [tw] OR “partner engagement” [tw] OR “male involvement” [tw] OR “husband involvement” [tw] OR “partner involvement” OR “male participation” [tw] OR “husband participation” [tw] OR “partner participation” [tw] OR “male involvement” [tw] OR “husband involvement” [tw] OR “partner involvement” [tw] OR “couple based” [tw] OR “couple involvement” [tw] OR “couple intervention” [tw] OR “spouse involvement” [tw] OR “spouse participation” [tw] OR “couples” [tw]

**Concept 3: LMICS**

Deprived Countries OR Deprived Population OR Deprived Populations OR Developing Countries OR Developing Country OR Developing Economies OR Developing Economy OR Developing Nation OR Developing Nations OR Developing Population OR Developing Populations OR Developing World OR LAMI Countries OR LAMI Country OR Less Developed Countries OR Less Developed Country OR Less Developed Economies OR Less Developed Nation OR Less Developed Nations OR Less Developed World OR Lesser Developed Countries OR Lesser Developed Nations OR LMIC OR LMICS OR Low GDP OR Low GNP OR Low Gross Domestic OR Low Gross National OR Low Income OR Lower GDP OR lower gross domestic OR Lower Income OR Middle Income OR Poor Countries OR Poor Country OR Poor Economies OR Poor Economy OR Poor Nation OR Poor Nations OR Poor Population OR Poor Populations OR poor world OR Poorer Countries OR Poorer Economies OR Poorer Economy OR Poorer Nations OR Poorer Population OR Poorer Populations OR Third World OR Transitional Countries OR Transitional Country OR Transitional Economies OR Transitional Economy OR Under Developed Countries OR Under Developed Country OR under developed nations OR Under Developed World OR Under Served Population OR Under Served Populations OR Underdeveloped Countries OR Underdeveloped Country OR underdeveloped economies OR underdeveloped nations OR underdeveloped population OR Underdeveloped World OR Underserved Countries OR Underserved Nations OR Underserved Population OR Underserved Populations OR Afghanistan OR Albania OR Algeria OR American Samoa OR Angola OR Armenia OR Azerbaijan OR Bangladesh OR Belarus OR Byelarus OR Belorussia OR Belize OR Benin OR Bhutan OR Bolivia OR Bosnia OR Botswana OR Brazil OR Bulgaria OR Burma OR Burkina Faso OR Burundi OR Cabo Verde OR Cape Verde OR Cambodia OR Cameroon OR Central African Republic OR Chad OR China OR Colombia OR Comoros OR Comores OR Comoro OR Congo OR Costa Rica OR Côte d'Ivoire OR Cuba OR Djibouti OR Dominica OR Dominican Republic OR Ecuador OR Egypt OR El Salvador OR Equatorial Guinea OR Eritrea OR Ethiopia OR Fiji OR Gabon OR Gambia OR Gaza OR Georgia OR Georgia Republic OR Ghana OR Grenada OR Grenadines OR Guatemala OR Guinea OR Guinea- Bissau OR Guyana OR Haiti OR Herzegovina OR Hercegovina OR Honduras OR India OR Indonesia OR Iran OR Iraq OR Ivory Coast OR Jamaica OR Jordan OR Kazakhstan OR Kenya OR Kiribati OR Democratic People’s Republic of Korea OR Kosovo OR Kyrgyz OR Kirghizia OR Kirghiz OR Kyrgyzstan OR Lao PDR OR Laos OR Lebanon OR Lesotho OR Liberia OR Libya OR Macedonia OR Madagascar OR Malawi OR Malay OR Malaya OR Malaysia OR Maldives OR Mali OR Marshall Islands OR Mauritania OR Mauritius OR Mexico OR Micronesia OR Moldova OR Mongolia OR Montenegro OR Morocco OR Mozambique OR Myanmar OR Namibia OR Nepal OR Nicaragua OR Niger OR Nigeria OR Pakistan OR Palau OR Papua New Guinea OR Paraguay OR Peru OR Philippines OR Principe OR Romania OR Ruanda OR Rwanda OR Samoa OR Sao Tome OR Senegal OR Serbia OR Sierra Leone OR Solomon Islands OR Somalia OR South Africa OR South Sudan OR Sri Lanka OR St Lucia OR St Vincent OR Sudan OR Surinam OR Suriname OR Swaziland OR Syria OR Syrian Arab Republic OR Tajikistan OR Tadzhikistan OR Tajikistan OR Tadzhik OR Tanzania OR Thailand OR Timor OR Togo OR Tonga OR Tunisia OR Turkey OR Turkmen OR Turkmenistan OR Tuvalu OR Uganda OR Ukraine OR Uzbek OR Uzbekistan OR Vanuatu OR Venezuela OR Vietnam OR West Bank OR Yemen OR Zambia OR Zimbabwe

**Concept 4: Not GBV/IPV**

“Violence” [Mesh] OR “Gender-Based Violence” [Mesh] OR “Intimate Partner Violence” [Mesh] OR “interpersonal violence” [tw] OR “gender based violence” [tw] OR “violence against women” [tw] OR “domestic violence” [tw]

**EMBASE**

**Concept 1: Women’s Health**

'women`s health'/exp OR 'reproductive health'/exp OR 'maternal care'/exp ('breast cancer' OR 'cervical cancer' OR obstetrics OR gynecology OR endometriosis OR menopause OR osteoporosis OR anemia OR thyroid OR 'menstrual health' OR 'female health' OR 'sexual health' OR 'prenatal care' OR 'antenatal care' OR 'womens screening' OR 'womens preventive care’ OR 'perintal depression’ OR ‘postpartum depression’ OR ‘postpartum’ OR ‘maternal mental health’):ti,ab,kw

**Concept 2: Male engagement**

('male engagement' OR 'husband engagement' OR 'partner engagement' OR 'male involvement' Or 'husband involvement' OR 'partner involvement' OR 'partner participation' OR 'male participation' OR 'husband participation' OR ‘couple based’ OR “couple involvement’ OR ‘couple intervention’ OR ‘spouse participation’ OR ‘couples’):ti,ab,kw

**Concept 3: LMICS**

("deprived countries":de,ti,ab OR "deprived country":de,ti,ab OR "deprived nation":de,ti,ab OR "deprived nations":de,ti,ab OR "deprived population":de,ti,ab OR "deprived populations":de,ti,ab OR "deprived world":de,ti,ab OR "developing countries":de,ti,ab OR "developing country":de,ti,ab OR "developing economies":de,ti,ab OR "developing economy":de,ti,ab OR "developing nation":de,ti,ab OR "developing nations":de,ti,ab OR "developing population":de,ti,ab OR "developing populations":de,ti,ab OR "developing world":de,ti,ab OR "lami countries":de,ti,ab OR "lami country":de,ti,ab OR "less developed countries":de,ti,ab OR "less developed country":de,ti,ab OR "less developed economies":de,ti,ab OR "less developed economy":de,ti,ab OR "less developed nation":de,ti,ab OR "less developed nations":de,ti,ab OR "less developed population":de,ti,ab OR "less developed populations":de,ti,ab OR "less developed world":de,ti,ab OR "lesser developed countries":de,ti,ab OR "lesser developed country":de,ti,ab OR "lesser developed economies":de,ti,ab OR "lesser developed economy":de,ti,ab OR "lesser developed nation":de,ti,ab OR "lesser developed nations":de,ti,ab OR "lesser developed population":de,ti,ab OR "lesser developed populations":de,ti,ab OR "lesser developed world":de,ti,ab OR "LMIC":de,ti,ab OR "LMICS":de,ti,ab OR "low gdp":de,ti,ab OR "low gnp":de,ti,ab OR "low gross domestic":de,ti,ab OR "low gross national":de,ti,ab OR "low income countries":de,ti,ab OR "low income country":de,ti,ab OR "low income economies":de,ti,ab OR "low income economy":de,ti,ab OR "low income nation":de,ti,ab OR "low income nations":de,ti,ab OR "low income population":de,ti,ab OR "low income populations":de,ti,ab OR "lower gdp":de,ti,ab OR "lower gnp":de,ti,ab OR "lower gross domestic":de,ti,ab OR "lower gross national":de,ti,ab OR "lower income countries":de,ti,ab OR "lower income country":de,ti,ab OR "lower income economies":de,ti,ab OR "lower income economy":de,ti,ab OR "lower income nation":de,ti,ab OR "lower income nations":de,ti,ab OR "lower income population":de,ti,ab OR "lower income populations":de,ti,ab OR "middle income countries":de,ti,ab OR "middle income country":de,ti,ab OR "middle income economies":de,ti,ab OR "middle income economy":de,ti,ab OR "middle income nation":de,ti,ab OR "middle income nations":de,ti,ab OR "middle income population":de,ti,ab OR "middle income populations":de,ti,ab OR "poor countries":de,ti,ab OR "poor country":de,ti,ab OR "Poor Economies":de,ti,ab OR "Poor Economy":de,ti,ab OR "poor nation":de,ti,ab OR "poor nations":de,ti,ab OR "poor population":de,ti,ab OR "poor populations":de,ti,ab OR "poor world":de,ti,ab OR "poorer countries":de,ti,ab OR "poorer country":de,ti,ab OR "Poorer Economies":de,ti,ab OR "Poorer Economy":de,ti,ab OR "poorer nation":de,ti,ab OR "poorer nations":de,ti,ab OR "poorer population":de,ti,ab OR "poorer populations":de,ti,ab OR "poorer world":de,ti,ab OR "third world":de,ti,ab OR "transitional countries":de,ti,ab OR "transitional country":de,ti,ab OR "Transitional Economies":de,ti,ab OR "Transitional Economy":de,ti,ab OR "under developed countries":de,ti,ab OR "under developed country":de,ti,ab OR "under developed economies":de,ti,ab OR "under developed economy":de,ti,ab OR "under developed nation":de,ti,ab OR "under developed nations":de,ti,ab OR "under developed population":de,ti,ab OR "under developed populations":de,ti,ab OR "under developed world":de,ti,ab OR "under served countries":de,ti,ab OR "under served country":de,ti,ab OR "under served nation":de,ti,ab OR "under served nations":de,ti,ab OR "under served population":de,ti,ab OR "under served populations":de,ti,ab OR "under served world":de,ti,ab OR "underdeveloped countries":de,ti,ab OR "underdeveloped country":de,ti,ab OR "underdeveloped economies":de,ti,ab OR "underdeveloped economy":de,ti,ab OR "underdeveloped nation":de,ti,ab OR "underdeveloped nations":de,ti,ab OR "underdeveloped population":de,ti,ab OR "underdeveloped populations":de,ti,ab OR "underdeveloped world":de,ti,ab OR "underserved countries":de,ti,ab OR "underserved country":de,ti,ab OR "underserved nation":de,ti,ab OR "underserved nations":de,ti,ab OR "underserved population":de,ti,ab OR "underserved populations":de,ti,ab OR "underserved world":de,ti,ab)

AND (Afghanistan:de,ti,ab OR Albania:de,ti,ab OR Algeria:de,ti,ab OR “American Samoa”:de,ti,ab OR Angola:de,ti,ab OR Argentina:de,ti,ab OR “Argentine Republic”:de,ti,ab OR Armenia:de,ti,ab OR Azerbaijan:de,ti,ab OR Bangladesh:de,ti,ab OR Belarus:de,ti,ab OR Byelarus:de,ti,ab OR Belorussia:de,ti,ab OR Belize:de,ti,ab OR Benin:de,ti,ab OR Bhutan:de,ti,ab OR Bolivia:de,ti,ab OR Bosnia:de,ti,ab OR Botswana:de,ti,ab OR Brazil:de,ti,ab OR Bulgaria:de,ti,ab OR Burma:de,ti,ab OR “Burkina Faso”:de,ti,ab OR Burundi:de,ti,ab OR “Cabo Verde”:de,ti,ab OR “Cape verde”:de,ti,ab OR Cambodia:de,ti,ab OR Cameroon:de,ti,ab OR “Central African Republic”:de,ti,ab OR Chad:de,ti,ab OR China:de,ti,ab OR Colombia:de,ti,ab OR Comoros:de,ti,ab OR Comores:de,ti,ab OR Comoro:de,ti,ab OR Congo:de,ti,ab OR “Costa Rica”:de,ti,ab OR “Cote d Ivoire”:de,ti,ab OR Cuba:de,ti,ab OR Djibouti:de,ti,ab OR Dominica:de,ti,ab OR “Dominican Republic”:de,ti,ab OR Ecuador:de,ti,ab OR Egypt:de,ti,ab OR “El Salvador”:de,ti,ab OR Eritrea:de,ti,ab OR Ethiopia:de,ti,ab OR Fiji:de,ti,ab OR Gabon:de,ti,ab OR Gambia:de,ti,ab OR Gaza:de,ti,ab OR “Georgia Republic”:de,ti,ab OR Georgian:de,ti,ab OR Ghana:de,ti,ab OR Grenada:de,ti,ab OR Grenadines:de,ti,ab OR Guatemala:de,ti,ab OR Guinea:de,ti,ab OR “Guinea Bissau”:de,ti,ab OR Guyana:de,ti,ab OR Haiti:de,ti,ab OR Herzegovina:de,ti,ab OR Hercegovina:de,ti,ab OR Honduras:de,ti,ab OR India:de,ti,ab OR Indonesia:de,ti,ab OR Iran:de,ti,ab OR Iraq:de,ti,ab OR Jamaica:de,ti,ab OR Jordan:de,ti,ab OR Kazakhstan:de,ti,ab OR Kenya:de,ti,ab OR Kiribati:de,ti,ab OR Korea:de,ti,ab OR Kosovo:de,ti,ab OR Kyrgyz:de,ti,ab OR Kirghizia:de,ti,ab OR Kirghiz:de,ti,ab OR Kirgizstan:de,ti,ab OR Kyrgyzstan:de,ti,ab OR “Lao PDR”:de,ti,ab OR Laos:de,ti,ab OR Lebanon:de,ti,ab OR Lesotho:de,ti,ab OR Liberia:de,ti,ab OR Libya:de,ti,ab OR Macedonia:de,ti,ab OR Madagascar:de,ti,ab OR Malawi:de,ti,ab OR Malay:de,ti,ab OR Malaya:de,ti,ab OR Malaysia:de,ti,ab OR Maldives:de,ti,ab OR Mali:de,ti,ab OR “Marshall Islands”:de,ti,ab OR Mauritania:de,ti,ab OR Mauritius:de,ti,ab OR Mexico:de,ti,ab OR Micronesia:de,ti,ab OR Moldova:de,ti,ab OR Mongolia:de,ti,ab OR Montenegro:de,ti,ab OR Morocco:de,ti,ab OR Mozambique:de,ti,ab OR Myanmar:de,ti,ab OR Namibia:de,ti,ab OR Nauru:de,ti,ab OR Nepal:de,ti,ab OR Nicaragua:de,ti,ab OR Niger:de,ti,ab OR Nigeria:de,ti,ab OR Pakistan:de,ti,ab OR Palau:de,ti,ab OR Panama:de,ti,ab OR “Papua New Guinea”:de,ti,ab OR Paraguay:de,ti,ab OR Peru:de,ti,ab OR Philippines:de,ti,ab OR Phillippines:de,ti,ab OR Philipines:de,ti,ab OR Phillipines:de,ti,ab OR Principe:de,ti,ab OR Romania:de,ti,ab OR Rwanda:de,ti,ab OR Ruanda:de,ti,ab OR Samoa:de,ti,ab OR “Sao Tome”:de,ti,ab OR Senegal:de,ti,ab OR Serbia:de,ti,ab OR “Sierra Leone”:de,ti,ab OR “Solomon Islands”:de,ti,ab OR Somalia:de,ti,ab OR “South Africa”:de,ti,ab OR “South Sudan”:de,ti,ab OR “Sri Lanka”:de,ti,ab OR “St Lucia”:de,ti,ab OR “St Vincent”:de,ti,ab OR Sudan:de,ti,ab OR Surinam:de,ti,ab OR Suriname:de,ti,ab OR Swaziland:de,ti,ab OR Syria:de,ti,ab OR “Syrian Arab Republic”:de,ti,ab OR Tajikistan:de,ti,ab OR Tadzhikistan:de,ti,ab OR Tadjikistan:de,ti,ab OR Tadzhik:de,ti,ab OR Tanzania:de,ti,ab OR Thailand:de,ti,ab OR Timor:de,ti,ab OR Togo:de,ti,ab OR Tonga:de,ti,ab OR Tunisia:de,ti,ab OR Turkey:de,ti,ab OR Turkmen:de,ti,ab OR Turkmenistan:de,ti,ab OR Tuvalu:de,ti,ab OR Uganda:de,ti,ab OR Ukraine:de,ti,ab OR Uzbek:de,ti,ab OR Uzbekistan:de,ti,ab OR Vanuatu:de,ti,ab OR Venezuela:de,ti,ab OR Vietnam:de,ti,ab OR “West Bank”:de,ti,ab OR Yemen:de,ti,ab OR Zambia:de,ti,ab OR Zimbabwe:de,ti,ab)

**Concept 4: Not GBV/IPV**

'gender based violence'/exp OR 'violence'/exp OR 'partner violence'/exp OR 'domestic violence'/exp

**PsychInfo**

**Concept 1: Women’s Health**

DE ( women's health or reproductive health or female health ) OR AB ( breast cancer OR cervical cancer OR obstetrics OR gynecology OR endometriosis OR menopause OR osteoporosis OR anemia OR thyroid OR menstrual health OR female health OR sexual health OR prenatal care OR antenatal care OR women’s screening OR women’s preventive care ) OR KW ( breast cancer OR cervical cancer OR obstetrics OR gynecology OR endometriosis OR menopause OR osteoporosis OR anemia OR thyroid OR menstrual health OR female health OR sexual health OR prenatal care OR antenatal care OR women’s screening OR women’s preventive care OR perinatal depression OR postpartum depression OR postpartum OR maternal mental health )

**Concept 2: Male engagement**

DE husband involvement OR AB ( male engagement OR husband engagement OR partner engagement OR male involvement OR husband involvement OR partner involvement OR male participation OR husband participation OR partner participation OR couple based OR couple involvement OR couple intervention OR spouse involvement OR spouse participation OR couples ) OR KW ( male engagement OR husband engagement OR partner engagement OR male involvement OR husband involvement OR partner involvement OR male participation OR husband participation OR partner participation OR couple based OR couple involvement OR couple intervention OR spouse involvement OR spouse participation OR couples )

**Concept 3: LMICS**

AB ( Afghanistan OR Albania OR Algeria OR “American Samoa” OR Angola OR Argentina OR “Argentine Republic” OR Armenia OR Azerbaijan OR Bangladesh OR Belarus OR Byelarus OR Belorussia OR Belize OR Benin OR Bhutan OR Bolivia OR Bosnia OR Botswana OR Brazil OR Bulgaria OR Burma OR “Burkina Faso” OR Burundi OR “Cabo Verde” OR “Cape verde” OR Cambodia OR Cameroon OR “Central African Republic” OR Chad OR China OR Colombia OR Comoros OR Comores OR Comoro OR Congo OR “Costa Rica” OR “Côte d'Ivoire” OR Cuba OR Djibouti OR Dominica OR “Dominican Republic” OR Ecuador OR Egypt OR “El Salvador” OR Eritrea OR Ethiopia OR Fiji OR Gabon OR Gambia OR Gaza OR “Georgia Republic” OR Georgian OR Ghana OR Grenada OR Grenadines OR Guatemala OR Guinea OR “Guinea Bissau” OR Guyana OR Haiti OR Herzegovina OR Hercegovina OR Honduras OR India OR Indonesia OR Iran OR Iraq OR Jamaica OR Jordan OR Kazakhstan OR Kenya OR Kiribati OR Korea OR Kosovo OR Kyrgyz OR Kirghizia OR Kirghiz OR Kirgizstan OR Kyrgyzstan OR “Lao PDR” OR Laos OR Lebanon OR Lesotho OR Liberia OR Libya OR Macedonia OR Madagascar OR Malawi OR Malay OR Malaya OR Malaysia OR Maldives OR Mali OR “Marshall Islands” OR Mauritania OR Mauritius OR Mexico OR Micronesia OR Moldova OR Mongolia OR Montenegro OR Morocco OR Mozambique OR Myanmar OR Namibia OR Nauru OR Nepal OR Nicaragua OR Niger OR Nigeria OR Pakistan OR Palau OR Panama OR “Papua New Guinea” OR Paraguay OR Peru OR Philippines OR Phillippines OR Philipines OR Phillipines OR Principe OR Romania OR Rwanda OR Ruanda OR Samoa OR “Sao Tome” OR Senegal OR Serbia OR “Sierra Leone” OR “Solomon Islands” OR Somalia OR “South Africa” OR “South Sudan” OR “Sri Lanka” OR “St Lucia” OR “St Vincent” OR Sudan OR Surinam OR Suriname OR Swaziland OR Syria OR “Syrian Arab Republic” OR Tajikistan OR Tadzhikistan OR Tadjikistan OR Tadzhik OR Tanzania OR Thailand OR Timor OR Togo OR Tonga OR Tunisia OR Turkey OR Turkmen OR Turkmenistan OR Tuvalu OR Uganda OR Ukraine OR Uzbek OR Uzbekistan OR Vanuatu OR Venezuela OR Vietnam OR “West Bank” OR Yemen OR Zambia OR Zimbabwe OR "deprived countries" OR "deprived country" OR "deprived nation" OR "deprived nations" OR "deprived population" OR "deprived populations" OR "deprived world" OR "developing countries" OR "developing country" OR "developing economies" OR "developing economy" OR "developing nation" OR "developing nations" OR "developing population" OR "developing populations" OR "developing world" OR "lami countries" OR "lami country" OR "less developed countries" OR "less developed country" OR "less developed economies" OR "less developed economy" OR "less developed nation" OR "less developed nations" OR "less developed population" OR "less developed populations" OR "less developed world" OR "lesser developed countries" OR "lesser developed country" OR "lesser developed economies" OR "lesser developed economy" OR "lesser developed nation" OR "lesser developed nations" OR "lesser developed population" OR "lesser developed populations" OR "lesser developed world" OR "LMIC" OR "LMICS" OR "low gdp" OR "low gnp" OR "low gross domestic" OR "low gross national" OR "low income countries" OR "low income country" OR "low income economies" OR "low income economy" OR "low income nation" OR "low income nations" OR "low income population" OR "low income populations" OR "lower gdp" OR "lower gnp" OR "lower gross domestic" OR "lower gross national" OR "lower income countries" OR "lower income country" OR "lower income economies" OR "lower income economy" OR "lower income nation" OR "lower income nations" OR "lower income population" OR "lower income populations" OR "middle income countries" OR "middle income country" OR "middle income economies" OR "middle income economy" OR "middle income nation" OR "middle income nations" OR "middle income population" OR "middle income populations" OR "poor countries" OR "poor country" OR "Poor Economies" OR "Poor Economy" OR "poor nation" OR "poor nations" OR "poor population" OR "poor populations" OR "poor world" OR "poorer countries" OR "poorer country" OR "Poorer Economies" OR "Poorer Economy" OR "poorer nation" OR "poorer nations" OR "poorer population" OR "poorer populations" OR "poorer world" OR "third world" OR "transitional countries" OR "transitional country" OR "Transitional Economies" OR "Transitional Economy" OR "under developed countries" OR "under developed country" OR "under developed economies" OR "under developed economy" OR "under developed nation" OR "under developed nations" OR "under developed population" OR "under developed populations" OR "under developed world" OR "under served countries" OR "under served country" OR "under served nation" OR "under served nations" OR "under served population" OR "under served populations" OR "under served world" OR "underdeveloped countries" OR "underdeveloped country" OR "underdeveloped economies" OR "underdeveloped economy" OR "underdeveloped nation" OR "underdeveloped nations" OR "underdeveloped population" OR "underdeveloped populations" OR "underdeveloped world" OR "underserved countries" OR "underserved country" OR "underserved nation" OR "underserved nations" OR "underserved population" OR "underserved populations" OR "underserved world" ) OR TI ( Afghanistan OR Albania OR Algeria OR “American Samoa” OR Angola OR Argentina OR “Argentine Republic” OR Armenia OR Azerbaijan OR Bangladesh OR Belarus OR Byelarus OR Belorussia OR Belize OR Benin OR Bhutan OR Bolivia OR Bosnia OR Botswana OR Brazil OR Bulgaria OR Burma OR “Burkina Faso” OR Burundi OR “Cabo Verde” OR “Cape verde” OR Cambodia OR Cameroon OR “Central African Republic” OR Chad OR China OR Colombia OR Comoros OR Comores OR Comoro OR Congo OR “Costa Rica” OR “Côte d'Ivoire” OR Cuba OR Djibouti OR Dominica OR “Dominican Republic” OR Ecuador OR Egypt OR “El Salvador” OR Eritrea OR Ethiopia OR Fiji OR Gabon OR Gambia OR Gaza OR “Georgia Republic” OR Georgian OR Ghana OR Grenada OR Grenadines OR Guatemala OR Guinea OR “Guinea Bissau” OR Guyana OR Haiti OR Herzegovina OR Hercegovina OR Honduras OR India OR Indonesia OR Iran OR Iraq OR Jamaica OR Jordan OR Kazakhstan OR Kenya OR Kiribati OR Korea OR Kosovo OR Kyrgyz OR Kirghizia OR Kirghiz OR Kirgizstan OR Kyrgyzstan OR “Lao PDR” OR Laos OR Lebanon OR Lesotho OR Liberia OR Libya OR Macedonia OR Madagascar OR Malawi OR Malay OR Malaya OR Malaysia OR Maldives OR Mali OR “Marshall Islands” OR Mauritania OR Mauritius OR Mexico OR Micronesia OR Moldova OR Mongolia OR Montenegro OR Morocco OR Mozambique OR Myanmar OR Namibia OR Nauru OR Nepal OR Nicaragua OR Niger OR Nigeria OR Pakistan OR Palau OR Panama OR “Papua New Guinea” OR Paraguay OR Peru OR Philippines OR Phillippines OR Philipines OR Phillipines OR Principe OR Romania OR Rwanda OR Ruanda OR Samoa OR “Sao Tome” OR Senegal OR Serbia OR “Sierra Leone” OR “Solomon Islands” OR Somalia OR “South Africa” OR “South Sudan” OR “Sri Lanka” OR “St Lucia” OR “St Vincent” OR Sudan OR Surinam OR Suriname OR Swaziland OR Syria OR “Syrian Arab Republic” OR Tajikistan OR Tadzhikistan OR Tadjikistan OR Tadzhik OR Tanzania OR Thailand OR Timor OR Togo OR Tonga OR Tunisia OR Turkey OR Turkmen OR Turkmenistan OR Tuvalu OR Uganda OR Ukraine OR Uzbek OR Uzbekistan OR Vanuatu OR Venezuela OR Vietnam OR “West Bank” OR Yemen OR Zambia OR Zimbabwe OR "deprived countries" OR "deprived country" OR "deprived nation" OR "deprived nations" OR "deprived population" OR "deprived populations" OR "deprived world" OR "developing countries" OR "developing country" OR "developing economies" OR "developing economy" OR "developing nation" OR "developing nations" OR "developing population" OR "developing populations" OR "developing world" OR "lami countries" OR "lami country" OR "less developed countries" OR "less developed country" OR "less developed economies" OR "less developed economy" OR "less developed nation" OR "less developed nations" OR "less developed population" OR "less developed populations" OR "less developed world" OR "lesser developed countries" OR "lesser developed country" OR "lesser developed economies" OR "lesser developed economy" OR "lesser developed nation" OR "lesser developed nations" OR "lesser developed population" OR "lesser developed populations" OR "lesser developed world" OR "LMIC" OR "LMICS" OR "low gdp" OR "low gnp" OR "low gross domestic" OR "low gross national" OR "low income countries" OR "low income country" OR "low income economies" OR "low income economy" OR "low income nation" OR "low income nations" OR "low income population" OR "low income populations" OR "lower gdp" OR "lower gnp" OR "lower gross domestic" OR "lower gross national" OR "lower income countries" OR "lower income country" OR "lower income economies" OR "lower income economy" OR "lower income nation" OR "lower income nations" OR "lower income population" OR "lower income populations" OR "middle income countries" OR "middle income country" OR "middle income economies" OR "middle income economy" OR "middle income nation" OR "middle income nations" OR "middle income population" OR "middle income populations" OR "poor countries" OR "poor country" OR "Poor Economies" OR "Poor Economy" OR "poor nation" OR "poor nations" OR "poor population" OR "poor populations" OR "poor world" OR "poorer countries" OR "poorer country" OR "Poorer Economies" OR "Poorer Economy" OR "poorer nation" OR "poorer nations" OR "poorer population" OR "poorer populations" OR "poorer world" OR "third world" OR "transitional countries" OR "transitional country" OR "Transitional Economies" OR "Transitional Economy" OR "under developed countries" OR "under developed country" OR "under developed economies" OR "under developed economy" OR "under developed nation" OR "under developed nations" OR "under developed population" OR "under developed populations" OR "under developed world" OR "under served countries" OR "under served country" OR "under served nation" OR "under served nations" OR "under served population" OR "under served populations" OR "under served world" OR "underdeveloped countries" OR "underdeveloped country" OR "underdeveloped economies" OR "underdeveloped economy" OR "underdeveloped nation" OR "underdeveloped nations" OR "underdeveloped population" OR "underdeveloped populations" OR "underdeveloped world" OR "underserved countries" OR "underserved country" OR "underserved nation" OR "underserved nations" OR "underserved population" OR "underserved populations" OR "underserved world" )

**Concept 4: Not GBV/IPV**

DE ( gender based violence or violence against women or intimate partner violence ) OR TX ( ipv or intimate partner violence or domestic violence or intimate partner aggression or partner abuse )

S1 AND S2 AND S3 NOT S4

**CINAHL**

**Concept 1: Women’s Health**

MH ( women's health or reproductive health or female health ) OR AB ( breast cancer OR cervical cancer OR obstetrics OR gynecology OR endometriosis OR menopause OR osteoporosis OR anemia OR thyroid OR menstrual health OR female health OR sexual health OR prenatal care OR antenatal care OR women’s screening OR women’s preventive care OR perinatal depression OR postpartum depression OR postpartum OR maternal mental health) OR TI (breast cancer OR cervical cancer OR obstetrics OR gynecology OR endometriosis OR menopause OR osteoporosis OR anemia OR thyroid OR menstrual health OR female health OR sexual health OR prenatal care OR antenatal care OR women’s screening OR women’s preventive care OR perinatal depression OR postpartum depression OR postpartum OR maternal mental health)

**Concept 2: Male engagement**

MH husband involvement OR AB ( male engagement OR husband engagement OR partner engagement OR male involvement OR husband involvement OR partner involvement OR male participation OR husband participation OR partner participation OR couple based OR couple involvement OR couple intervention OR spouse involvement OR spouse participation OR couples ) OR TI ( male engagement OR husband engagement OR partner engagement OR male involvement OR husband involvement OR partner involvement OR male participation OR husband participation OR partner participation OR couple based OR couple involvement OR couple intervention OR spouse involvement OR spouse participation OR couples )

**Concept 3: LMIC**

AB ( Afghanistan OR Albania OR Algeria OR “American Samoa” OR Angola OR Argentina OR “Argentine Republic” OR Armenia OR Azerbaijan OR Bangladesh OR Belarus OR Byelarus OR Belorussia OR Belize OR Benin OR Bhutan OR Bolivia OR Bosnia OR Botswana OR Brazil OR Bulgaria OR Burma OR “Burkina Faso” OR Burundi OR “Cabo Verde” OR “Cape verde” OR Cambodia OR Cameroon OR “Central African Republic” OR Chad OR China OR Colombia OR Comoros OR Comores OR Comoro OR Congo OR “Costa Rica” OR “Côte d'Ivoire” OR Cuba OR Djibouti OR Dominica OR “Dominican Republic” OR Ecuador OR Egypt OR “El Salvador” OR Eritrea OR Ethiopia OR Fiji OR Gabon OR Gambia OR Gaza OR “Georgia Republic” OR Georgian OR Ghana OR Grenada OR Grenadines OR Guatemala OR Guinea OR “Guinea Bissau” OR Guyana OR Haiti OR Herzegovina OR Hercegovina OR Honduras OR India OR Indonesia OR Iran OR Iraq OR Jamaica OR Jordan OR Kazakhstan OR Kenya OR Kiribati OR Korea OR Kosovo OR Kyrgyz OR Kirghizia OR Kirghiz OR Kirgizstan OR Kyrgyzstan OR “Lao PDR” OR Laos OR Lebanon OR Lesotho OR Liberia OR Libya OR Macedonia OR Madagascar OR Malawi OR Malay OR Malaya OR Malaysia OR Maldives OR Mali OR “Marshall Islands” OR Mauritania OR Mauritius OR Mexico OR Micronesia OR Moldova OR Mongolia OR Montenegro OR Morocco OR Mozambique OR Myanmar OR Namibia OR Nauru OR Nepal OR Nicaragua OR Niger OR Nigeria OR Pakistan OR Palau OR Panama OR “Papua New Guinea” OR Paraguay OR Peru OR Philippines OR Phillippines OR Philipines OR Phillipines OR Principe OR Romania OR Rwanda OR Ruanda OR Samoa OR “Sao Tome” OR Senegal OR Serbia OR “Sierra Leone” OR “Solomon Islands” OR Somalia OR “South Africa” OR “South Sudan” OR “Sri Lanka” OR “St Lucia” OR “St Vincent” OR Sudan OR Surinam OR Suriname OR Swaziland OR Syria OR “Syrian Arab Republic” OR Tajikistan OR Tadzhikistan OR Tadjikistan OR Tadzhik OR Tanzania OR Thailand OR Timor OR Togo OR Tonga OR Tunisia OR Turkey OR Turkmen OR Turkmenistan OR Tuvalu OR Uganda OR Ukraine OR Uzbek OR Uzbekistan OR Vanuatu OR Venezuela OR Vietnam OR “West Bank” OR Yemen OR Zambia OR Zimbabwe OR "deprived countries" OR "deprived country" OR "deprived nation" OR "deprived nations" OR "deprived population" OR "deprived populations" OR "deprived world" OR "developing countries" OR "developing country" OR "developing economies" OR "developing economy" OR "developing nation" OR "developing nations" OR "developing population" OR "developing populations" OR "developing world" OR "lami countries" OR "lami country" OR "less developed countries" OR "less developed country" OR "less developed economies" OR "less developed economy" OR "less developed nation" OR "less developed nations" OR "less developed population" OR "less developed populations" OR "less developed world" OR "lesser developed countries" OR "lesser developed country" OR "lesser developed economies" OR "lesser developed economy" OR "lesser developed nation" OR "lesser developed nations" OR "lesser developed population" OR "lesser developed populations" OR "lesser developed world" OR "LMIC" OR "LMICS" OR "low gdp" OR "low gnp" OR "low gross domestic" OR "low gross national" OR "low income countries" OR "low income country" OR "low income economies" OR "low income economy" OR "low income nation" OR "low income nations" OR "low income population" OR "low income populations" OR "lower gdp" OR "lower gnp" OR "lower gross domestic" OR "lower gross national" OR "lower income countries" OR "lower income country" OR "lower income economies" OR "lower income economy" OR "lower income nation" OR "lower income nations" OR "lower income population" OR "lower income populations" OR "middle income countries" OR "middle income country" OR "middle income economies" OR "middle income economy" OR "middle income nation" OR "middle income nations" OR "middle income population" OR "middle income populations" OR "poor countries" OR "poor country" OR "Poor Economies" OR "Poor Economy" OR "poor nation" OR "poor nations" OR "poor population" OR "poor populations" OR "poor world" OR "poorer countries" OR "poorer country" OR "Poorer Economies" OR "Poorer Economy" OR "poorer nation" OR "poorer nations" OR "poorer population" OR "poorer populations" OR "poorer world" OR "third world" OR "transitional countries" OR "transitional country" OR "Transitional Economies" OR "Transitional Economy" OR "under developed countries" OR "under developed country" OR "under developed economies" OR "under developed economy" OR "under developed nation" OR "under developed nations" OR "under developed population" OR "under developed populations" OR "under developed world" OR "under served countries" OR "under served country" OR "under served nation" OR "under served nations" OR "under served population" OR "under served populations" OR "under served world" OR "underdeveloped countries" OR "underdeveloped country" OR "underdeveloped economies" OR "underdeveloped economy" OR "underdeveloped nation" OR "underdeveloped nations" OR "underdeveloped population" OR "underdeveloped populations" OR "underdeveloped world" OR "underserved countries" OR "underserved country" OR "underserved nation" OR "underserved nations" OR "underserved population" OR "underserved populations" OR "underserved world" ) OR TI ( Afghanistan OR Albania OR Algeria OR “American Samoa” OR Angola OR Argentina OR “Argentine Republic” OR Armenia OR Azerbaijan OR Bangladesh OR Belarus OR Byelarus OR Belorussia OR Belize OR Benin OR Bhutan OR Bolivia OR Bosnia OR Botswana OR Brazil OR Bulgaria OR Burma OR “Burkina Faso” OR Burundi OR “Cabo Verde” OR “Cape verde” OR Cambodia OR Cameroon OR “Central African Republic” OR Chad OR China OR Colombia OR Comoros OR Comores OR Comoro OR Congo OR “Costa Rica” OR “Côte d'Ivoire” OR Cuba OR Djibouti OR Dominica OR “Dominican Republic” OR Ecuador OR Egypt OR “El Salvador” OR Eritrea OR Ethiopia OR Fiji OR Gabon OR Gambia OR Gaza OR “Georgia Republic” OR Georgian OR Ghana OR Grenada OR Grenadines OR Guatemala OR Guinea OR “Guinea Bissau” OR Guyana OR Haiti OR Herzegovina OR Hercegovina OR Honduras OR India OR Indonesia OR Iran OR Iraq OR Jamaica OR Jordan OR Kazakhstan OR Kenya OR Kiribati OR Korea OR Kosovo OR Kyrgyz OR Kirghizia OR Kirghiz OR Kirgizstan OR Kyrgyzstan OR “Lao PDR” OR Laos OR Lebanon OR Lesotho OR Liberia OR Libya OR Macedonia OR Madagascar OR Malawi OR Malay OR Malaya OR Malaysia OR Maldives OR Mali OR “Marshall Islands” OR Mauritania OR Mauritius OR Mexico OR Micronesia OR Moldova OR Mongolia OR Montenegro OR Morocco OR Mozambique OR Myanmar OR Namibia OR Nauru OR Nepal OR Nicaragua OR Niger OR Nigeria OR Pakistan OR Palau OR Panama OR “Papua New Guinea” OR Paraguay OR Peru OR Philippines OR Phillippines OR Philipines OR Phillipines OR Principe OR Romania OR Rwanda OR Ruanda OR Samoa OR “Sao Tome” OR Senegal OR Serbia OR “Sierra Leone” OR “Solomon Islands” OR Somalia OR “South Africa” OR “South Sudan” OR “Sri Lanka” OR “St Lucia” OR “St Vincent” OR Sudan OR Surinam OR Suriname OR Swaziland OR Syria OR “Syrian Arab Republic” OR Tajikistan OR Tadzhikistan OR Tadjikistan OR Tadzhik OR Tanzania OR Thailand OR Timor OR Togo OR Tonga OR Tunisia OR Turkey OR Turkmen OR Turkmenistan OR Tuvalu OR Uganda OR Ukraine OR Uzbek OR Uzbekistan OR Vanuatu OR Venezuela OR Vietnam OR “West Bank” OR Yemen OR Zambia OR Zimbabwe OR "deprived countries" OR "deprived country" OR "deprived nation" OR "deprived nations" OR "deprived population" OR "deprived populations" OR "deprived world" OR "developing countries" OR "developing country" OR "developing economies" OR "developing economy" OR "developing nation" OR "developing nations" OR "developing population" OR "developing populations" OR "developing world" OR "lami countries" OR "lami country" OR "less developed countries" OR "less developed country" OR "less developed economies" OR "less developed economy" OR "less developed nation" OR "less developed nations" OR "less developed population" OR "less developed populations" OR "less developed world" OR "lesser developed countries" OR "lesser developed country" OR "lesser developed economies" OR "lesser developed economy" OR "lesser developed nation" OR "lesser developed nations" OR "lesser developed population" OR "lesser developed populations" OR "lesser developed world" OR "LMIC" OR "LMICS" OR "low gdp" OR "low gnp" OR "low gross domestic" OR "low gross national" OR "low income countries" OR "low income country" OR "low income economies" OR "low income economy" OR "low income nation" OR "low income nations" OR "low income population" OR "low income populations" OR "lower gdp" OR "lower gnp" OR "lower gross domestic" OR "lower gross national" OR "lower income countries" OR "lower income country" OR "lower income economies" OR "lower income economy" OR "lower income nation" OR "lower income nations" OR "lower income population" OR "lower income populations" OR "middle income countries" OR "middle income country" OR "middle income economies" OR "middle income economy" OR "middle income nation" OR "middle income nations" OR "middle income population" OR "middle income populations" OR "poor countries" OR "poor country" OR "Poor Economies" OR "Poor Economy" OR "poor nation" OR "poor nations" OR "poor population" OR "poor populations" OR "poor world" OR "poorer countries" OR "poorer country" OR "Poorer Economies" OR "Poorer Economy" OR "poorer nation" OR "poorer nations" OR "poorer population" OR "poorer populations" OR "poorer world" OR "third world" OR "transitional countries" OR "transitional country" OR "Transitional Economies" OR "Transitional Economy" OR "under developed countries" OR "under developed country" OR "under developed economies" OR "under developed economy" OR "under developed nation" OR "under developed nations" OR "under developed population" OR "under developed populations" OR "under developed world" OR "under served countries" OR "under served country" OR "under served nation" OR "under served nations" OR "under served population" OR "under served populations" OR "under served world" OR "underdeveloped countries" OR "underdeveloped country" OR "underdeveloped economies" OR "underdeveloped economy" OR "underdeveloped nation" OR "underdeveloped nations" OR "underdeveloped population" OR "underdeveloped populations" OR "underdeveloped world" OR "underserved countries" OR "underserved country" OR "underserved nation" OR "underserved nations" OR "underserved population" OR "underserved populations" OR "underserved world" )

**Concept 4: Not GBV/IPV**

DE ( gender based violence or violence against women or intimate partner violence ) OR AB ( ipv or intimate partner violence or domestic violence or intimate partner aggression or partner abuse )

S1 AND S2 AND S3 NOT S4

**SCOPUS**

**Concept 1: Women’s’ health**

TITLE-ABS-KEY ( "women health" OR "reproductive health" OR "maternal health" OR "women’s " OR "breast cancer" OR "cervical cancer" OR "obstetrics" OR "gynecology" OR "endometriosis" OR "menopause" OR "osteoporosis" OR "anemia" OR "thyroid" OR "menstrual health" OR "women* health" OR "female health" OR "sexual health" OR "prenatal care" OR "antenatal care" OR "women* screening" OR "women* preventive care" OR "perinatal depression" OR "postpartum depression" OR "postpartum" OR "maternal mental health" )

**Concept 2: Male engagement**

TITLE-ABS-KEY ( “male engagement” OR “husband engagement” OR “partner engagement” OR “male involvement” OR “husband involvement” OR “partner involvement” OR “male participation” OR “husband participation” OR “partner participation” OR “couple based” OR “couple involvement” OR “couple intervention” OR “spouse involvement” OR “spouse participation” OR “couples”)

**Concept 3: LMIC**

TITLE-ABS-KEY ( afghanistan OR albania OR algeria OR "American Samoa" OR angola OR armenia OR azerbaijan OR bangladesh OR belarus OR byelarus OR belorussia OR belize OR benin OR bhutan OR bolivia OR bosnia OR botswana OR brazil OR bulgaria OR burma OR "Burkina Faso" OR burundi OR "Cabo Verde" OR "Cape Verde" OR cambodia OR cameroon OR "Central African Republic" OR chad OR china OR colombia OR comoros OR comores OR comoro OR congo OR "Costa Rica" OR "Côte d'Ivoire" OR cuba OR "Democratic People’s Republic of Korea" OR djibouti OR dominica OR "Dominican Republic" OR ecuador OR egypt OR "El Salvador" OR eritrea OR ethiopia OR "Equatorial Guinea" OR fiji OR gabon OR gambia OR gaza OR "Georgia Republic" OR georgia OR ghana OR grenada OR grenadines OR guatemala OR guinea OR "Guinea Bissau" OR guyana OR haiti OR herzegovina OR hercegovina OR honduras OR india OR indonesia OR iran OR iraq OR "Ivory Coast" OR jamaica OR jordan OR kazakhstan OR kenya OR kiribati OR korea OR kosovo OR kyrgyz OR kirghizia OR kirghiz OR kyrgyzstan OR "Lao PDR" OR laos OR lebanon OR lesotho OR liberia OR libya OR macedonia OR madagascar OR malawi OR malay OR malaya OR malaysia OR maldives OR mali OR "Marshall Islands" OR mauritania OR mauritius OR mexico OR micronesia OR moldova OR mongolia OR montenegro OR morocco OR mozambique OR myanmar OR namibia OR nepal AND ornicaragua OR niger OR nigeria OR pakistan OR palau OR papua AND new AND guinea OR paraguay OR peru OR philippines OR principe OR romania OR rwanda OR ruanda OR samoa OR "Sao Tome" OR senegal OR serbia OR "Sierra Leone" OR "Solomon Islands" OR somalia OR "South Africa" OR "South Sudan" OR "Sri Lanka" OR "St Lucia" OR "St Vincent" OR sudan OR surinam OR suriname OR swaziland OR syria OR "Syrian Arab Republic" OR tajikistan OR tadzhikistan OR tajikistan OR tadzhik OR tanzania OR thailand OR timor OR togo OR tonga OR tunisia OR turkey OR turkmen OR turkmenistan OR tuvalu OR uganda OR ukraine OR uzbek OR uzbekistan OR vanuatu OR venezuela OR vietnam OR "West Bank" OR yemen OR zambia OR zimbabwe )

TITLE-ABS-KEY ( "Deprived Countries" OR "Deprived Population" OR "Deprived Populations" OR "Developing Countries" OR "Developing Country" OR "Developing Economies" OR "Developing Economy" OR "Developing Nation" OR "Developing Nations" OR "Developing Population" OR "Developing Populations" OR "Developing World" OR "LAMI Countries" OR "LAMI Country" OR "Less Developed Countries" OR "Less Developed Country" OR "Less Developed Economies" OR "Less Developed Nation" OR "Less Developed Nations" OR "Less Developed World" OR "Lesser Developed Countries" OR "Lesser Developed Nations" OR lmic OR lmics OR "Low GDP" OR "Low GNP" OR "Low Gross Domestic" OR "Low Gross National" OR "Low Income" OR "Lower income" OR "Lower GDP" OR "Lower Gross Domestic" OR "Middle Income" OR "Poor Countries" OR "Poor Country" OR "Poor Economies" OR "Poor Economy" OR "Poor Nation" OR "Poor Nations" OR "Poor Population" OR "Poor Populations" OR "poor world" OR "Poorer Countries" OR "Poorer Economies" OR "Poorer Economy" OR "Poorer Nations" OR "Poorer Population" OR "Poorer Populations" OR "Third World" OR "Transitional Countries" OR "Transitional Country" OR "Transitional Economies" OR "Transitional Economy" OR "Under Developed" OR "Under Served" OR "Underdeveloped Countries" OR "Underdeveloped Country" OR "underdeveloped economies" OR "underdeveloped nations" OR "underdeveloped population" OR "Underdeveloped World" OR "Underserved Countries" OR "Underserved Nations" OR "Underserved Population" OR "Underserved Populations" )

**Concept 4: GBV**

TITLE-ABS-KEY ( "interpersonal violence" OR "gender based violence" OR "violence against women" OR "domestic violence" OR "violence" OR "marital conflict" OR "conflict resolution" )

( TITLE-ABS-KEY ( "Deprived Countries" OR "Deprived Population" OR "Deprived Populations" OR "Developing Countries" OR "Developing Country" OR "Developing Economies" OR "Developing Economy" OR "Developing Nation" OR "Developing Nations" OR "Developing Population" OR "Developing Populations" OR "Developing World" OR "LAMI Countries" OR "LAMI Country" OR "Less Developed Countries" OR "Less Developed Country" OR "Less Developed Economies" OR "Less Developed Nation" OR "Less Developed Nations" OR "Less Developed World" OR "Lesser Developed Countries" OR "Lesser Developed Nations" OR lmic OR lmics OR "Low GDP" OR "Low GNP" OR "Low Gross Domestic" OR "Low Gross National" OR "Low Income" OR "Lower income" OR "Lower GDP" OR "Lower Gross Domestic" OR "Middle Income" OR "Poor Countries" OR "Poor Country" OR "Poor Economies" OR "Poor Economy" OR "Poor Nation" OR "Poor Nations" OR "Poor Population" OR "Poor Populations" OR "poor world" OR "Poorer Countries" OR "Poorer Economies" OR "Poorer Economy" OR "Poorer Nations" OR "Poorer Population" OR "Poorer Populations" OR "Third World" OR "Transitional Countries" OR "Transitional Country" OR "Transitional Economies" OR "Transitional Economy" OR "Under Developed" OR "Under Served" OR "Underdeveloped Countries" OR "Underdeveloped Country" OR "underdeveloped economies" OR "underdeveloped nations" OR "underdeveloped population" OR "Underdeveloped World" OR "Underserved Countries" OR "Underserved Nations" OR "Underserved Population" OR "Underserved Populations" ) ) OR ( TITLE-ABS-KEY ( afghanistan OR albania OR algeria OR "American Samoa" OR angola OR armenia OR azerbaijan OR bangladesh OR belarus OR byelarus OR belorussia OR belize OR benin OR bhutan OR bolivia OR bosnia OR botswana OR brazil OR bulgaria OR burma OR "Burkina Faso" OR burundi OR "Cabo Verde" OR "Cape Verde" OR cambodia OR cameroon OR "Central African Republic" OR chad OR china OR colombia OR comoros OR comores OR comoro OR congo OR "Costa Rica" OR "Côte d'Ivoire" OR cuba OR "Democratic People’s Republic of Korea" OR djibouti OR dominica OR "Dominican Republic" OR ecuador OR egypt OR "El Salvador" OR eritrea OR ethiopia OR "Equatorial Guinea" OR fiji OR gabon OR gambia OR gaza OR "Georgia Republic" OR georgia OR ghana OR grenada OR grenadines OR guatemala OR guinea OR "Guinea Bissau" OR guyana OR haiti OR herzegovina OR hercegovina OR honduras OR india OR indonesia OR iran OR iraq OR "Ivory Coast" OR jamaica OR jordan OR kazakhstan OR kenya OR kiribati OR korea OR kosovo OR kyrgyz OR kirghizia OR kirghiz OR kyrgyzstan OR "Lao PDR" OR laos OR lebanon OR lesotho OR liberia OR libya OR macedonia OR madagascar OR malawi OR malay OR malaya OR malaysia OR maldives OR mali OR "Marshall Islands" OR mauritania OR mauritius OR mexico OR micronesia OR moldova OR mongolia OR montenegro OR morocco OR mozambique OR myanmar OR namibia OR nepal AND ornicaragua OR niger OR nigeria OR pakistan OR palau OR papua AND new AND guinea OR paraguay OR peru OR philippines OR principe OR romania OR rwanda OR ruanda OR samoa OR "Sao Tome" OR senegal OR serbia OR "Sierra Leone" OR "Solomon Islands" OR somalia OR "South Africa" OR "South Sudan" OR "Sri Lanka" OR "St Lucia" OR "St Vincent" OR sudan OR surinam OR suriname OR swaziland OR syria OR "Syrian Arab Republic" OR tajikistan OR tadzhikistan OR tajikistan OR tadzhik OR tanzania OR thailand OR timor OR togo OR tonga OR tunisia OR turkey OR turkmen OR turkmenistan OR tuvalu OR uganda OR ukraine OR uzbek OR uzbekistan OR vanuatu OR venezuela OR vietnam OR "West Bank" OR yemen OR zambia OR zimbabwe ) ) AND ( TITLE-ABS-KEY ( "women health" OR "reproductive health" OR "maternal health" OR "women&apos;s" OR "breast cancer" OR "cervical cancer" OR "obstetrics" OR "gynecology" OR "endometriosis" OR "menopause" OR "osteoporosis" OR "anemia" OR "thyroid" OR "menstrual health" OR "women* health" OR "female health" OR "sexual health" OR "prenatal care" OR "antenatal care" OR "women* screening" OR "women* preventive care" OR "perinatal depression" OR "postpartum depression" OR "postpartum" OR "maternal mental health" ) ) AND ( TITLE-ABS-KEY ( "male engagement" OR "husband engagement" OR "partner engagement" OR "male involvement" OR "husband involvement" OR "partner involvement" OR "male participation" OR "husband participation" OR "partner participation" OR "couple based" OR "couple involvement" OR "couple intervention" OR "spouse involvement" OR "spouse participation" OR "couples" ) ) AND NOT ( TITLE-ABS-KEY ( "interpersonal violence" OR "gender based violence" OR "violence against women" OR "domestic violence" OR "violence" OR "marital conflict" OR "conflict resolution" ) )

**WHO Global Index Medicus**

tw:(( mh:("Reproductive Health" OR "Maternal Health" OR "Postpartum Period" OR "Prenatal Care")) tw:((tw:("women health" OR "reproductive health" OR "maternal health" OR "women’s " OR "breast cancer" OR "cervical cancer" OR "obstetrics" OR "gynecology" OR "endometriosis" OR "menopause" OR "osteoporosis" OR "anemia" OR "thyroid" OR "menstrual health" OR "women* health" OR "female health" OR "sexual health" OR "prenatal care" OR "antenatal care" OR "women* screening" OR "women* preventive care" OR "perinatal depression" OR "postpartum depression" OR "postpartum" OR "maternal mental health" )) AND (tw:(( “male engagement” OR “husband engagement” OR “partner engagement” OR “male involvement” OR “husband involvement” OR “partner involvement” OR “male participation” OR “husband participation” OR “partner participation” OR “couple based” OR “couple involvement” OR “couple intervention” OR “spouse involvement” OR “spouse participation” OR “couples”))))) AND ( la:("en"))

IBSS

**Concept 1: Womens health**

summary(“women health” OR “reproductive health” OR “maternal health” OR "women's" OR "breast cancer" OR "cervical cancer" OR "obstetrics" OR "gynecology" OR "endometriosis" OR "menopause" OR "osteoporosis" OR "anemia" OR "thyroid" OR "menstrual health" OR "women's health" OR "female health" OR "sexual health" OR "prenatal care" OR “antenatal care” OR "women's screening" OR "women's preventive care" OR “perinatal depression” OR “postpartum depression” OR “postpartum” OR “maternal mental health” )

**Concept 2: Male engagement**

summary(“male engagement” OR “husband engagement” OR “partner engagement” OR “male involvement” OR “husband involvement” OR “partner involvement” OR “male participation” OR “husband participation” OR “partner participation” OR “couple based” OR “couple involvement” OR “couple intervention” OR “spouse involvement” OR “spouse participation” OR “couples”)

**Concept 3: LMIC**

[summary(Deprived Countries OR Deprived Population OR Deprived Populations OR Developing Countries OR Developing Country OR Developing Economies OR Developing Economy OR Developing Nation OR Developing Nations OR Developing Population OR Developing Populations OR Developing World OR LAMI Countries OR LAMI Country OR Less Developed Countries OR Less Developed Country OR Less Developed Economies OR Less Developed Nation OR Less Developed Nations OR Less Developed World OR Lesser Developed Countries OR Lesser Developed Nations OR LMIC OR LMICS OR Low GDP OR Low GNP OR Low Gross Domestic OR Low Gross National OR Low Income OR Lower GDP OR lower gross domestic OR Lower Income OR Middle Income OR Poor Countries OR Poor Country OR Poor Economies OR Poor Economy OR Poor Nation OR Poor Nations OR Poor Population OR Poor Populations OR poor world OR Poorer Countries OR Poorer Economies OR Poorer Economy OR Poorer Nations OR Poorer Population OR Poorer Populations OR Third World OR Transitional Countries OR Transitional Country OR Transitional Economies OR Transitional Economy OR Under Developed Countries OR Under Developed Country OR under developed nations OR Under Developed World OR Under Served Population OR Under Served Populations OR Underdeveloped Countries OR Underdeveloped Country OR underdeveloped economies OR underdeveloped nations OR underdeveloped population OR Underdeveloped World OR Underserved Countries OR Underserved Nations OR Underserved Population OR Underserved Populations OR Afghanistan OR Albania OR Algeria OR American Samoa OR Angola OR Armenia OR Azerbaijan OR Bangladesh OR Belarus OR Byelarus OR Belorussia OR Belize OR Benin OR Bhutan OR Bolivia OR Bosnia OR Botswana OR Brazil OR Bulgaria OR Burma OR Burkina Faso OR Burundi OR Cabo Verde OR Cape Verde OR Cambodia OR Cameroon OR Central African Republic OR Chad OR China OR Colombia OR Comoros OR Comores OR Comoro OR Congo OR Costa Rica OR Côte d'Ivoire OR Cuba OR Democratic People’s Republic of Korea OR Djibouti OR Dominica OR Dominican Republic OR Ecuador OR Egypt OR El Salvador OR Equatorial Guinea OR Eritrea OR Ethiopia OR Fiji OR Gabon OR Gambia OR Gaza OR Georgia OR Georgia Republic OR Ghana OR Grenada OR Grenadines OR Guatemala OR Guinea OR Guinea Bissau OR Guyana OR Haiti OR Herzegovina OR Hercegovina OR Honduras OR India OR Indonesia OR Iran OR Iraq OR Ivory Coast OR Jamaica OR Jordan OR Kazakhstan OR Kenya OR Kiribati OR Korea OR Kosovo OR Kyrgyz OR Kirghizia OR Kirghiz OR Kyrgyzstan OR Lao PDR OR Laos OR Lebanon OR Lesotho OR Liberia OR Libya OR Macedonia OR Madagascar OR Malawi OR Malay OR Malaya OR Malaysia OR Maldives OR Mali OR Marshall Islands OR Mauritania OR Mauritius OR Mexico OR Micronesia OR Moldova OR Mongolia OR Montenegro OR Morocco OR Mozambique OR Myanmar OR Namibia OR Nepal OR Nicaragua OR Niger OR Nigeria OR Pakistan OR Palau OR Papua New Guinea OR Paraguay OR Peru OR Philippines OR Principe OR Romania OR Rwanda OR Ruanda OR Samoa OR Sao Tome OR Senegal OR Serbia OR Sierra Leone OR Solomon Islands OR Somalia OR South Africa OR South Sudan OR Sri Lanka OR St Lucia OR St Vincent OR Sudan OR Surinam OR Suriname OR Swaziland OR Syria OR Syrian Arab Republic OR Tajikistan OR Tadzhikistan OR Tajikistan OR Tadzhik OR Tanzania OR Thailand OR Timor OR Togo OR Tonga OR Tunisia OR Turkey OR Turkmen OR Turkmenistan OR Tuvalu OR Uganda OR Ukraine OR Uzbek OR Uzbekistan OR Vanuatu OR Venezuela OR Vietnam OR West Bank OR Yemen OR Zambia OR Zimbabwe)](https://www.proquest.com/recentsearches.recentsearchtabview.recentsearchesgridview.scrolledrecentsearchlist.checkdbssearchlink:rerunsearch/B04FC08BB82848DBPQ/None?site=ibss&t:ac=RecentSearches)

**Concept 4: NOT GBV**

summary("interpersonal violence” OR “gender based violence” OR “violence against women” OR “domestic violence” OR “violence” OR “marital conflict” OR “conflict resolution”)

Cochrane

Concept 1: Women’s health

Concept 2: male engagement

Concept 3: LMICS

Concept 4: NOT GBV

Cochrane reviews

## 13 - (("maternal health" OR "maternal health" “maternal health” OR "women's" OR "breast cancer" OR "cervical cancer" OR "obstetrics" OR "gynecology" OR "endometriosis" OR "menopause" OR "osteoporosis" OR "anemia" OR "thyroid" OR "menstrual health" OR "women's health" OR "female health" OR "sexual health" OR "prenatal care" OR “antenatal care” OR "women's screening" OR "women's preventive care" OR “perinatal depression” OR “postpartum depression” OR “postpartum” OR “maternal mental health”) AND (“male engagement” OR “husband engagement” OR “partner engagement” OR “male involvement” OR “husband involvement” OR “partner involvement” OR “male participation” OR “husband participation” OR “partner participation” OR “couple based” OR “couple involvement” OR “couple intervention” OR “spouse involvement” OR “spouse participation”)):ti,ab,kw"

**Proquest Dissertations and Theses Global**

**Concept 1: Women’s health**

summary(“women health” OR “reproductive health” OR “maternal health” OR "women's" OR "breast cancer" OR "cervical cancer" OR "obstetrics" OR "gynecology" OR "endometriosis" OR "menopause" OR "osteoporosis" OR "anemia" OR "thyroid" OR "menstrual health" OR "women's health" OR "female health" OR "sexual health" OR "prenatal care" OR “antenatal care” OR "women's screening" OR "women's preventive care" OR “perinatal depression” OR “postpartum depression” OR “postpartum” OR “maternal mental health” )

**Concept 2: Male engagement**

summary(“male engagement” OR “husband engagement” OR “partner engagement” OR “male involvement” OR “husband involvement” OR “partner involvement” OR “male participation” OR “husband participation” OR “partner participation” OR “couple based” OR “couple involvement” OR “couple intervention” OR “spouse involvement” OR “spouse participation” OR “couples”)

**Concept 3: LMIC**

[summary(Deprived Countries OR Deprived Population OR Deprived Populations OR Developing Countries OR Developing Country OR Developing Economies OR Developing Economy OR Developing Nation OR Developing Nations OR Developing Population OR Developing Populations OR Developing World OR LAMI Countries OR LAMI Country OR Less Developed Countries OR Less Developed Country OR Less Developed Economies OR Less Developed Nation OR Less Developed Nations OR Less Developed World OR Lesser Developed Countries OR Lesser Developed Nations OR LMIC OR LMICS OR Low GDP OR Low GNP OR Low Gross Domestic OR Low Gross National OR Low Income OR Lower GDP OR lower gross domestic OR Lower Income OR Middle Income OR Poor Countries OR Poor Country OR Poor Economies OR Poor Economy OR Poor Nation OR Poor Nations OR Poor Population OR Poor Populations OR poor world OR Poorer Countries OR Poorer Economies OR Poorer Economy OR Poorer Nations OR Poorer Population OR Poorer Populations OR Third World OR Transitional Countries OR Transitional Country OR Transitional Economies OR Transitional Economy OR Under Developed Countries OR Under Developed Country OR under developed nations OR Under Developed World OR Under Served Population OR Under Served Populations OR Underdeveloped Countries OR Underdeveloped Country OR underdeveloped economies OR underdeveloped nations OR underdeveloped population OR Underdeveloped World OR Underserved Countries OR Underserved Nations OR Underserved Population OR Underserved Populations Afghanistan OR Albania OR Algeria OR American Samoa OR Angola OR Armenia OR Azerbaijan OR Bangladesh OR Belarus OR Byelarus OR Belorussia OR Belize OR Benin OR Bhutan OR Bolivia OR Bosnia OR Botswana OR Brazil OR Bulgaria OR Burma OR Burkina Faso OR Burundi OR Cabo Verde OR Cape Verde OR Cambodia OR Cameroon OR Central African Republic OR Chad OR China OR Colombia OR Comoros OR Comores OR Comoro OR Congo OR Costa Rica OR Côte d'Ivoire OR Cuba OR Democratic People’s Republic of Korea OR Djibouti OR Dominica OR Dominican Republic OR Ecuador OR Egypt OR El Salvador OR Equatorial Guinea OR Eritrea OR Ethiopia OR Fiji OR Gabon OR Gambia OR Gaza OR Georgia OR Georgia Republic OR Ghana OR Grenada OR Grenadines OR Guatemala OR Guinea OR Guinea Bissau OR Guyana OR Haiti OR Herzegovina OR Hercegovina OR Honduras OR India OR Indonesia OR Iran OR Iraq OR Ivory Coast OR Jamaica OR Jordan OR Kazakhstan OR Kenya OR Kiribati OR Korea OR Kosovo OR Kyrgyz OR Kirghizia OR Kirghiz OR Kyrgyzstan OR Lao PDR OR Laos OR Lebanon OR Lesotho OR Liberia OR Libya OR Macedonia OR Madagascar OR Malawi OR Malay OR Malaya OR Malaysia OR Maldives OR Mali OR Marshall Islands OR Mauritania OR Mauritius OR Mexico OR Micronesia OR Moldova OR Mongolia OR Montenegro OR Morocco OR Mozambique OR Myanmar OR Namibia OR Nepal OR Nicaragua OR Niger OR Nigeria OR Pakistan OR Palau OR Papua New Guinea OR Paraguay OR Peru OR Philippines OR Principe OR Romania OR Rwanda OR Ruanda OR Samoa OR Sao Tome OR Senegal OR Serbia OR Sierra Leone OR Solomon Islands OR Somalia OR South Africa OR South Sudan OR Sri Lanka OR St Lucia OR St Vincent OR Sudan OR Surinam OR Suriname OR Swaziland OR Syria OR Syrian Arab Republic OR Tajikistan OR Tadzhikistan OR Tajikistan OR Tadzhik OR Tanzania OR Thailand OR Timor OR Togo OR Tonga OR Tunisia OR Turkey OR Turkmen OR Turkmenistan OR Tuvalu OR Uganda OR Ukraine OR Uzbek OR Uzbekistan OR Vanuatu OR Venezuela OR Vietnam OR West Bank OR Yemen OR Zambia OR Zimbabwe)](https://www.proquest.com/recentsearches.recentsearchtabview.recentsearchesgridview.scrolledrecentsearchlist.checkdbssearchlink:rerunsearch/B04FC08BB82848DBPQ/None?site=ibss&t:ac=RecentSearches)

**Concept 4: NOT GBV**

summary("interpersonal violence” OR “gender based violence” OR “violence against women” OR “domestic violence” OR “violence” OR “marital conflict” OR “conflict resolution”)

**Global Health Observatory (GHO)**

Maternal and reproductive health and male engagement

Women’s health and male engagement

**The Communication Initiative Network**

"male engagement" "women's health" "maternal health" mental

**USAID DEC**

With the words “male engagement” “women’s health” “mental health”

“male engagement” “maternal health” “mental health”

“husband engagement” “women’s health” “mental”

“male engagement” “reproductive health” “mental”

“male engagement” “postpartum” “mental health”

“male engagement” “mental health”

“male engagement” “mental wellbeing”

With words “male engagement”

Must include one of – mental health, mental, depression, anxiety, psychosocial

**UK department for international development**

“male engagement” “women’s health” “mental health”

“male engagement” “women’s health “

“male engagement” “women’ health”

“male engagement” “maternal”

“husband engagement”

"partner involvement" "mental health"

"postpartum" "mental health" "male engagement"

"perinatal" "mental health" "male engagement"

OAIster

kw:("women's health") AND kw:("male engagement") AND kw:("mental health")

su:("women's health") AND su:("male engagement") AND su:("mental health")

su:("maternal health") AND su:("male engagement") AND su:("mental health")

su:("maternal health") AND su:("male engagement") AND su:("mental wellbeing")

su:("women's health") AND su:("male engagement") AND su:("depression")

su:("women's health") AND su:("male engagement") AND su:("anxiety")

kw:("women's health") AND kw:("male engagement") AND kw:("anxiety")

kw:("women's health") AND kw:("male engagement") AND kw:("stress")

su:("women's health") AND su:("male engagement") AND su:("stress")

su:("women's health") AND su:("couple based") AND su:("stress")

su:("women's health") AND su:("couple") AND su:("mental health")

Clinical trials . gov

Condition / disease (“women health” OR “reproductive health” OR “maternal health” OR "women's" OR "breast cancer" OR "cervical cancer" OR "obstetrics" OR "gynecology" OR "endometriosis" OR "menopause" OR "osteoporosis" OR "anemia" OR "thyroid" OR "menstrual health" OR "women's health" OR "female health" OR "sexual health" OR "prenatal care" OR “antenatal care” OR "women's screening" OR "women's preventive care" OR “perinatal depression” OR “postpartum depression” OR “postpartum” OR “maternal mental health” )

Other terms: (“male engagement” OR “husband engagement” OR “partner engagement” OR “male involvement” OR “husband involvement” OR “partner involvement” OR “male participation” OR “husband participation” OR “partner participation” OR “couple based” OR “couple involvement” OR “couple intervention” OR “spouse involvement” OR “spouse participation” OR “couples”)

Outcome measure "mental health" OR "depression" OR "mental" OR "anxiety" OR "stress" OR “PTSD”

Age range 15 years or older

Intervention

- Not yet recruiting or recruiting
- Recruiting
- Active not recruiting
- Complete
- Terminated

International Clinical Trial Registry Platform

(("maternal health" OR "maternal health" “maternal health” OR "women's" OR "breast cancer" OR "cervical cancer" OR "obstetrics" OR "gynecology" OR "endometriosis" OR "menopause" OR "osteoporosis" OR "anemia" OR "thyroid" OR "menstrual health" OR "women's health" OR "female health" OR "sexual health" OR "prenatal care" OR “antenatal care” OR "women's screening" OR "women's preventive care" OR “perinatal depression” OR “postpartum depression” OR “postpartum” OR “maternal mental health”) AND (“male engagement” OR “husband engagement” OR “partner engagement” OR “male involvement” OR “husband involvement” OR “partner involvement” OR “male participation” OR “husband participation” OR “partner participation” OR “couple based” OR “couple involvement” OR “couple intervention” OR “spouse involvement” OR “spouse participation”)):ti,ab,kw

ON Cochrane CENTRAL
