# Supplementary material for: The mental health impact on women of engaging men in health interventions in low- and middle-income countries: A systematic review
Source: PLOS Glob Public Health. 2025 Nov 3;5(11):e0005168. doi: 10.1371/journal.pgph.0005168 (PMC12582469; doi:10.1371/journal.pgph.0005168)
Supplement: S1 Table — (DOCX) [file pgph.0005168.s003.docx]

S1 Table. Quality Assessment Using the JBI Critical Appraisal Checklists

| **Citation - 1st author's last name and year** | **Study Design** | **Quality Assessment Tool Used** | **Questions** | | | | | | | | | | | | |
| --- | --- | --- | --- | --- | --- | --- | --- | --- | --- | --- | --- | --- | --- | --- | --- |
|  |  | **JBI Critical Appraisal Checklist for RCT** | **1-true randomization** | **2-concealed allocation** | **3-similar groups at baseline** | **4-participants blinded** | **5-those delivering intervention blinded** | **6-treatment groups were treated identically other than intervention** | **7-outcome assessors blind to treatment assignment** | **8-outcomes measured in the same way** | **9-outcomes measure in a reliable way** | **10-was follow up complete** | **11-were participants analyzed in the groups to which they were randomized** | **12-appropriate statistical analysis used** | **13-trial design was appropriate and deviations from RCT were account for in the conduct and analysis** |
| **Akbarian 2018** | RCT |  | Yes | Yes | Yes | No | No | Yes | No | Yes | Yes | Yes | Yes | Yes | Yes |
| **Comrie-Thomson 2022** | cRCT |  | Yes | Yes | Yes | No | No | Yes | No | Yes | Yes | Yes | Yes | Yes | Yes |
| **Fourianalistyawati 2023** | RCT |  | Yes | Yes | Yes | No | N/A | Yes | No | Yes | Yes | Yes | Yes | Yes | Yes |
| **Jones 2018, Jones 2021, Abbamonte 2021, Peltzer 2020, Sifunda 2019** | cRCT |  | Yes | Yes | Yes | No | No | Yes | No | Yes | Yes | Yes | Yes | Yes | Yes |
| **Maitra 2017** | RCT |  | Yes | Yes | Yes | No | No | Yes | No | Yes | Yes | Yes | Yes | N/A | Yes |
| **Mosalanejad 2013** | RCT |  | Yes | Yes | Yes | No | No | Yes | No | Yes | Yes | Yes | Yes | Yes | Yes |
| **Rabiepoor 2019** | RCT |  | Yes | Yes | Yes | No | No | Yes | No | Yes | Yes | Yes | Yes | Yes | Yes |
| **Sorkhani 2021** | RCT |  | Yes | Yes | Yes | No | No | Yes | No | Yes | Yes | Yes | Yes | Yes | Yes |
| **Sulaiman 2022** | RCT |  | Yes | Yes | Yes | No | N/A | Yes | No | Yes | Yes | Yes | Yes | Yes | Yes |
| **Villar-Loubet 2013** | RCT |  | Yes | Yes | Yes | No | No | Yes | No | No | Yes | Yes | Yes | N/A | Yes |
|  |  | **JBI Critical Appraisal Checklist for Quasi-Experimental Studies** | **1-it is clear what the “cause” and “effect” is (i.e. no confusion about which variable comes first)** | **2- was there a control group?** | **3-were participants included in any comparisons similar to each other?** | **4-were participants included in any comparisons receiving similar treatment/care** | **5-were there multiple measurements of the outcome** | **6-outcomes of participants included in any comparisons measured in the same way** | **7-outcomes were measured in a reliable way** | **8-was follow up complete** | **9-appropritae statistical analysis used** |  |  |  |  |
| **Çömez 2020** | Quasi-experimental study |  | Yes | Yes | Yes | Yes | Yes | Yes | Yes | Yes | Yes |  |  |  |  |
| **Dehshiri 2023** | Quasi-experimental study |  | Yes | Yes | Yes | Yes | Yes | Yes | Yes | Yes | Yes |  |  |  |  |
| **Sayari 2022** | Quasi-experimental study |  | Yes | Yes | Yes | Yes | Yes | Yes | Yes | Yes | Yes |  |  |  |  |
| **Mindry 2018** | Non-experimental one arm study |  | Yes | No | N/A | N/A | No | N/A | Yes | Yes | N/A |  |  |  |  |
